# Facioscapulohumeral dystrophy transcriptome signatures correlate with different stages of disease and are marked by different MRI biomarkers

**DOI:** 10.1038/s41598-022-04817-8

**Published:** 2022-01-26

**Authors:** Anita van den Heuvel, Saskia Lassche, Karlien Mul, Anna Greco, David San León Granado, Arend Heerschap, Benno Küsters, Stephen J. Tapscott, Nicol C. Voermans, Baziel G. M. van Engelen, Silvère M. van der Maarel

**Affiliations:** 1 Department of Human Genetics, Leiden University Medical Center, Albinusdreef 2, Postal zone S-04-P, 2333 ZA Leiden, The Netherlands; 2 Department of Neurology, Zuyderland Medical Center, Heerlen, The Netherlands; 3 Department of Neurology, Donders Institute for Brain, Cognition and Behaviour, Radboud University Medical Center, Nijmegen, The Netherlands; 4 Department of Systems Biology, National Center of Biotechnology (CNB-CSIC), Madrid, Spain; 5 Department of Radiology, Radboud University Medical Center, Nijmegen, The Netherlands; 6 Department of Pathology, Radboud University Medical Center, Nijmegen, The Netherlands; 7 Division of Human Biology, Fred Hutchinson Cancer Research Center, Seattle, WA 98109 USA

**Keywords:** Biomarkers, Magnetic resonance imaging, Neuromuscular disease

## Abstract

With several therapeutic strategies for facioscapulohumeral muscular dystrophy (FSHD) entering clinical testing, outcome measures are becoming increasingly important. Considering the spatiotemporal nature of FSHD disease activity, clinical trials would benefit from non-invasive imaging-based biomarkers that can predict FSHD-associated transcriptome changes. This study investigated two FSHD-associated transcriptome signatures (DUX4 and PAX7 signatures) in FSHD skeletal muscle biopsies, and tested their correlation with a variety of disease-associated factors, including Ricci clinical severity score, disease duration, D4Z4 repeat size, muscle pathology scorings and functional outcome measures. It establishes that DUX4 and PAX7 signatures both show a sporadic expression pattern in FSHD-affected biopsies, possibly marking different stages of disease. This study analyzed two imaging-based biomarkers—Turbo Inversion Recovery Magnitude (TIRM) hyperintensity and fat fraction—and provides insights into their predictive power as non-invasive biomarkers for FSHD signature detection in clinical trials. Further insights in the heterogeneity of—and correlation between—imaging biomarkers and molecular biomarkers, as provided in this study, will provide important guidance to clinical trial design in FSHD. Finally, this study investigated the role of infiltrating non-muscle cell types in FSHD signature expression and detected potential distinct roles for two fibro-adipogenic progenitor subtypes in FSHD.

## Introduction

With a prevalence of 12/100,000 individuals, facioscapulohumeral muscular dystrophy (FSHD) is one of the most prevalent hereditary skeletal muscle disorders in adults^1^. Patients suffer from progressive weakness of the muscles of the face, shoulders and upper arms. In more advanced disease muscle weakness extends to the trunk, pelvic girdle and lower limbs, often with severe asymmetrical foot drop^2^. About 20% of FSHD patients become wheelchair-dependent by age 50^3,4^. FSHD is caused by mis-expression in skeletal muscle of the double homeobox 4 (*DUX4*) gene from the D4Z4 macrosatellite repeat in the subtelomere of chromosome 4 (4q35), either due to D4Z4 repeat contractions to 1–10 units or due to mutations in D4Z4 chromatin modifiers^5–8^. DUX4 is a germline and cleavage stage transcription factor and its expression in skeletal muscle elicits a transcriptional response (the DUX4 signature) that is unfamiliar to skeletal muscle eventually resulting in cell death^9–17^.

FSHD is hallmarked by considerable heterogeneity, both in transcriptome signatures, as in age at onset, disease penetrance, progression and severity even within families^18,19^. Myogenic cell cultures show sporadic and temporal bursts of DUX4 signature expression^20,21^ and in vivo, DUX4 signature expression can only be detected in approximately 60–70% of muscle biopsies^22,23^. How such sporadic DUX4 signature expression relates to the focal patterns of muscle pathology observed by MRI-based imaging remains partly understood. Also, how DUX4 signature expression relates to disease severity and progression remains to be investigated^21,22,24,25^. In addition, due to its competitive relation with DUX4, a PAX7-associated gene expression signature has been described in FSHD. Indeed a reduction in PAX7 score has been associated with increased disease pathology and advanced disease progression^26,27^. How this PAX7 signature relates to the focal nature of DUX4 expression and disease activity remains, however, to be determined. Because of the spatiotemporal nature of FSHD-associated transcriptome signatures, studies on FSHD disease progression would strongly benefit from non-invasive imaging-based biomarkers that can predict molecular signature expression.

In this study, we analyzed DUX4 and PAX7 signature expression in 39 FSHD and 24 control vastus lateralis (VL) or tibialis anterior (TA) muscle biopsies and studied their correlation with MRI-based biomarkers Turbo Inversion Recovery Magnitude (TIRM) hyperintensity and quantitative fat fraction. Hyperintensity changes in TIRM imaging are considered to reflect FSHD disease activity, being associated with higher levels of DUX4 signature expression, faster rates of progression of fatty infiltration and more severe histopathological abnormalities, including necrosis, regeneration and inflammation^22,28–34^. Fatty infiltration is considered to be the destructive consequence of DUX4-induced muscle damage and reflects FSHD disease severity, being correlated with clinical severity scores, functional performance and progression over time^29,31,35–39^. MRI studies have suggested that once fatty infiltration is present, disease progression is relatively rapid until the muscle is almost completely affected. However, whether fat fraction can be used as additional biomarker for regions with active FSHD signature expression remains to be determined.

Our data shows that DUX4 and PAX7 signatures are partially overlapping biomarkers for FSHD, both displaying a sporadic expression pattern in FSHD-affected biopsies and—based on correlation with imaging-based biomarkers—each representing different states of disease activity and/or progression. This suggests the utility of combining the biomarkers TIRM hyperintensity and fat fraction for increased detection of FSHD-associated transcriptional changes in clinical trials. Furthermore, we analyzed the role of infiltrating non-muscle cells in FSHD and identified potentially distinct roles for two subtypes of fibro-adipogenic progenitor cells in either DUX4 signature expression or PAX7 score reduction, respectively.

## Results

### Participants and muscle biopsies

We included 12 control and 28 genetically confirmed FSHD participants who did not differ in age, sex distribution or BMI (see Table 1 for summary metrics). In FSHD participants, the mean disease duration was 20.04 years and their median Ricci clinical severity score (CSS) was 6 (range 0–8). FSHD participants had impaired functional performance as measured with the six-minute walking test (6-MWT), the Motor Function Measure (MFM) and the Medical Research Council (MRC) score (Table 1). From these 40 participants, we obtained 63 muscle biopsies. We collected VL muscle biopsies from 26/28 FSHD participants and all control individuals (38 VL biopsies; control N = 12, FSHD N = 26). VL muscle biopsies from two FSHD participants were excluded from this study due to poor tissue quality or poor quality of the isolated RNA. In addition, eleven FSHD participants and all control individuals donated a second TA muscle biopsy (23 TA biopsies; control N = 12, FSHD N = 11). In two FSHD participants (FSHD-09_VL and FSHD-13_VL), MRI-guided biopsy enabled us to obtain two separate biopsies, one from a TIRM-positive (TIRM^POS^) and one from a TIRM-negative (TIRM^NEG^) area within the same VL muscle (Table 1 and Supplementary Table S1 for complete overview). Histopathology sum scoring on 49/63 muscle biopsies showed an expected elevated median histopathology sum score in FSHD (median sum score ± IQR of 4 ± 3, range 1–11) compared to control biopsies (median sum score ± IQR of 2 ± 2, range 0–4) (Table 1 and Supplementary Table S1). Separate evaluation for inflammatory infiltrates showed increased signs of inflammation in FSHD samples (Table 1 and Supplementary Table S1).

**Table 1:** Participant and muscle biopsy characteristics.

Participant-specific information							
		Unique participants only [All samples]					
Data	What values	CTRL		FSHD		p-value	Statistical test^e^
**n**		24		39			
**Sex**	%Male	50.00 [50.00]		57.14 [53.85]		0.74 [0.80]	Fisher’s exact-test
**Age**	Avg & sem	53.83 [53.83]	1.66 [1.15]	50.25 [51.10]	2.42 [1.77]	0.23 [0.20]	Student’s t-test
**BMI**	Avg & sem	27.34 [27.34]	1.53 [1.06]	24.55 [24.65]	0.62 [0.55]	0.11 [**0.031**]	Student’s t-test
**Repeat_units**^a^	Median & IQR	0		7.00 [7.00]	3.00 [3.00]		
1	# samples	0 [0]		0 [0]			
2		0 [0]		0 [0]			
3		0 [0]		2 [2]			
4		0 [0]		0 [0]			
5		0 [0]		8 [8]			
6		0 [0]		1 [2]			
7		0 [0]		3 [5]			
8		0 [0]		6 [8]			
9		0 [0]		2 [3]			
10		0 [0]		1 [2]			
FSHD2		0 [0]		4 [7]			
Mosaic 2units		0 [0]		1 [2]			
No FSHD		12 [24]		0 [0]			
**Ricci_CSS**	Median & IQR	NA	NA	6.00 [6.00]	4.25 [4.00]		
CTRL	# samples	12 [24]		0 [0]			
0		0 [0]		2 [2]			
1		0 [0]		1 [1]			
2		0 [0]		4 [6]			
3		0 [0]		3 [4]			
4		0 [0]		2 [3]			
5		0 [0]		1 [2]			
6		0 [0]		7 [9]			
7		0 [0]		4 [5]			
8		0 [0]		4 [7]			
9		0 [0]		0 [0]			
10		0 [0]		0 [0]			
**Disease duration (years)**^b^	Avg & sem	NA	NA	20.04 [22.79]	2.76 [2.34]		
**SixMWT**^c^	Avg & sem	513.50 [513.50]	13.04 [9.02]	448.19 [428.37]	24.33 [21.63]	**0.023 [0.00068]**	Student’s t-test
**MFM**	Median & IQR	0.99 [0.99]	0.01 [0.01]	0.92 [0.91]	0.15 [0.16]	**0.0090 [0.00012]**	Mann–Whitney U test

Sample-specific information (Participant and muscle-specific)							
		All samples					
Data	What values	CTRL		FSHD		p-value	Statistical test^e^
**Fat_percentage**	Avg & sem	5.36	1.12	18.49	4.12	**0.017**	Mann–Whitney U test
**TIRM**	# Pos	NA		5		0.15	Fisher's exact test
**MRC**	Median & IQR	5.00	0.00	5.00	0.75	**0.0020**	Fisher’s exact-test
0.0	# samples	0		1			
0.5		0		0			
1.0		0		0			
1.5		0		0			
2.0		0		2			
2.5		0		3			
3.0		0		0			
3.5		0		1			
4.0		0		3			
4.5		0		2			
5.0		24		27			
**Histology sum score**^d^	Median & IQR	2.00	2.00	4.00	3.00	**0.000055**	Mann–Whitney *U* test
0	# samples	2		0			
1		8		3			
2		7		3			
3		6		3			
4		1		7			
5		0		1			
6		0		2			
7		0		2			
8		0		2			
9		0		0			
10		0		1			
11		0		1			
NA		0		14			
**Inflammation score**^d^	Median & IQR	0.00	0.00	0.00	1.00	**0.023**	Fisher’s exact-test
0	# samples	23		17			
1		1		7			
*2*		0		1			
NA		0		14			
**DUX4 signature**	Avg & sem	6.44	0.69	85.36	28.43	**0.0010**	Student's t-test
**PAX7 score**	Avg & sem	− 6.89	0.16	− 7.31	0.17	0.071	Student's t-test

Significant values are in bold.

*BMI* Body mass index, *FSHD* facioscapulohumeral muscular dystrophy, *CSS* Clinical severity score, *6MWT* 6-min walking test, *MFM* Motor Function Measure, *MRC score* Medical Research Council, *VL* vastus lateralis, *TA* tibialis anterior.

^a^Excluding four FSHD2 participants caused by an SMCHD1 mutation & one participant who was mosaic for FSHD1 and had a 2-unit D4Z4 repeat in 65% of leukocytes.

^b^Including 2 asymptomatic cases.

^c^Excluding 1 patient who was not able to walk for 6 min.

^d^Excluding 14 FSHD samples without scoring.

^e^All student's T-tests are Welch-corrected for unequal variance.

– For the MRC score: Fisher’s exact test for normal score (MRC score = 5.0) versus reduced score (MRC < 5.0).

– For the Inflammation score: Fisher’s exact test for no inflammation (score = 0) versus signs of inflammation (score > 0).

– For DUX4 signature statistics, signature is log-transformed: log_10_[value + 1].

– For consistency between subgroup analyses.

– For Age, BMI and SixMWT, we used a parametric test data for the 'all samples' comparison even though data was not normally distributed in this comparison. This did not affect significance scoring for Age and SixMWT. BMI turned non-significant in a Mann–Whitney test. See also Supplementary Table S2 for the muscle type-specific results for all comparisons of this table.

### Imaging-based biomarker detection

**Figure 1:**
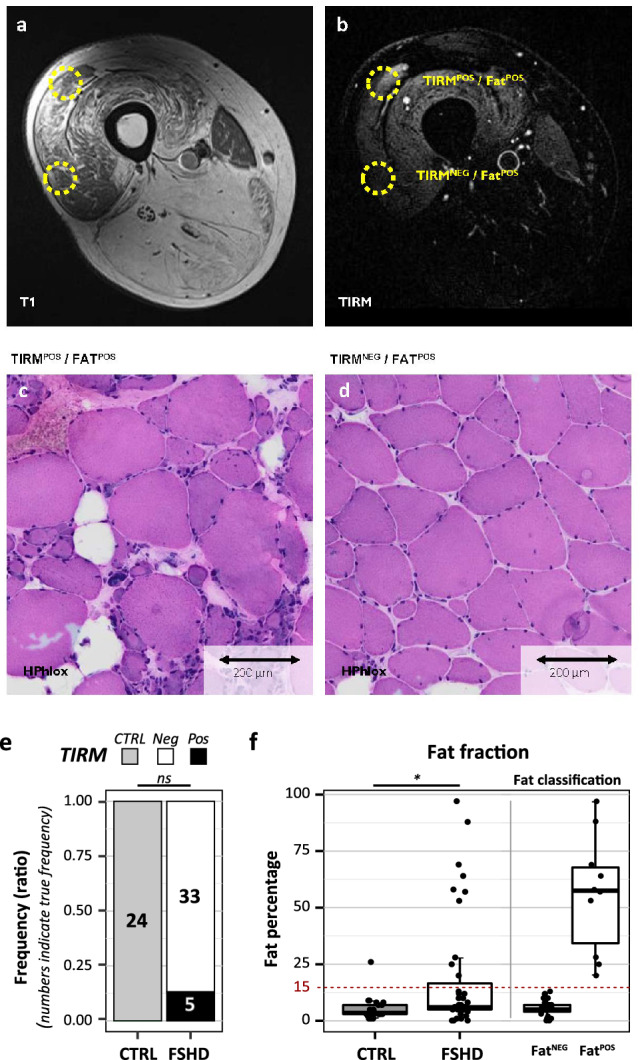
Representative examples of the two MRI-based imaging biomarkers used in this study; fatty infiltration and TIRM hyperintensity. (**a**) Axial T1-weighted and (**b**) TIRM image of the left upper leg of a 50-year-old FSHD patient (FSHD-09) showing marked fatty infiltration of nearly all muscles and focal hyperintensity in the VL muscle. The MRI-guided biopsy sites are marked with the yellow circles. In T1: Normal muscle is dark grey, fat infiltrated muscle is white. Note the relative sparing of the sartorius muscle and the severe fatty infiltration of the posterior compartment and quadriceps. (**c**) HPhlox staining of the Fat^POS^/TIRM^POS^ biopsy from the same patient demonstrates severe dystrophic changes indicated by a marked increase in fiber size variability, increased internal nuclei, regenerating fibers and fatty infiltration. (**d**) HPhlox staining of the Fat^POS^/TIRM^NEG^ biopsy from the same patient shows fiber size variability, increased internal nuclei and few regenerating fibers corresponding to mild dystrophic changes. (**e**) TIRM hyperintensity frequencies in muscle biopsies of each disease state. p-values depict a Fisher’s exact test result (excluding the replicate samples). (**f**) Fat fractions of all individual muscle biopsies grouped by disease state. p-values depict a Mann–Whitney *U* test results (excluding the replicate samples). The red dashed line indicates the classification threshold for Fat^POS^ versus Fat^NEG^ FSHD muscle biopsies (15% fat fraction) and the boxplots on the right depict the FSHD biopsies separated based on this classification. p-values: ns = not significant, *p < 0.05, **p < 0.01, ***p < 0.001, ****p < 0.0001. MRI﻿ scans were exported from Agfa IMPAX (https://global.agfahealthcare.com). Histology sections were digitized using a Philips UFS (www.usa.philips.com/healthcare/resources/landing/philips-intellisite-pathology-solution) and images were assessed using 3DHISTECH’s CaseViewer (v2.3, www.3dhistech.com). All data plots are generated in R (v4.0.3, www.R-project.org) using the packages *gplots* (v3.1.1) and *ggplot2* (v3.3.3). Figure and panel layout was further adapted in Adobe Illustrator CC 2018 (www.adobe.com).

Prior to biopsy, TIRM sequences and transversal T1-weighted, multi-echo T2 images or Dixon sequences were acquired of the upper and lower leg (Supplementary Table S1). Representative MRI and histopathology images of TIRM^POS^ muscle biopsies are provided in Fig. 1 and Supplementary Fig. S1. The multi-echo T2 images or Dixon fat fraction maps were then used to calculate muscle fat fractions.

Our biopsy cohort included five biopsies from a TIRM^POS^ muscle, all originating from FSHD-affected participants (Fig. 1e). This detection frequency is comparable to previously reported frequencies of TIRM hyperintensity in VL and TA muscles^35^.

Fat fraction was generally elevated in FSHD muscle biopsies compared to control biopsies (Table 1, Fig. 1f) and the FSHD biopsy cohort included ten biopsies with a fat fraction > 15%. Based on previous studies in healthy controls we labeled these biopsies as fat-positive (Fat^POS^), whereas all except for one control biopsies had a fat fraction ≤ 15% (Fig. 1f)^31,36,40,41^. Again, this frequency of Fat^POS^ FSHD biopsies is similar to previously reported frequencies of elevated fat fractions in the VL and TA muscles of FSHD patients^35^. Of note, most fat fractions were determined with multi-echo T2, though some VL fat fractions were measured with 3-pt Dixon (Supplementary Table S1). In our experience, quantitative analysis based on Dixon images overestimates fat fraction relative to analysis based on T2 images at low fat fractions. This may be important when comparing fat fractions of this study with literature. Though, all muscles evaluated by 3-pt Dixon had < 15% fat fraction, except for one participant (25%). Hence, the different imaging techniques most likely did not influence classification of muscle biopsies.

### FSHD-associated signature expression

**Figure 2:**
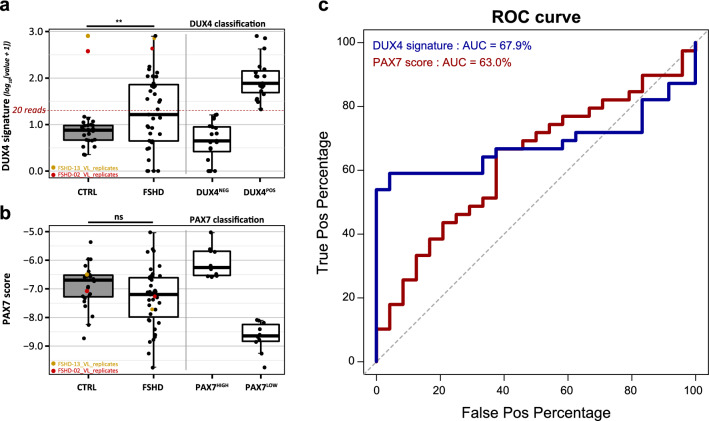
DUX4 and PAX7 signature expression in FSHD and control biopsies. (**a**) DUX4 signature expression in FSHD and control muscle biopsies. The threshold criterium for DUX4^POS^ biopsy selection (cumulative normalized read count > 20) is marked with a red dashed line and the boxplots on the right depict the FSHD biopsies separated based on this classification. The two replicate FSHD samples sequenced in the two major sequence batches are highlighted in color. Both replicates are selected as DUX4^POS^ in both sequence batches, indicating that the absence of a DUX4 signature in the controls is not due to a sequencing bias. (**b**) PAX7 signature expression in FSHD and control muscle biopsies. As a classification threshold for PAX7-affected versus non-affected biopsies could not be clearly defined based on the scores in control samples, for further subgroup comparisons the ten FSHD samples with the highest PAX7 score were classified as PAX7^HIGH^ (i.e. likely non-affected) and the ten FSHD samples with lowest PAX7 scores were classified as PAX7^LOW^ (i.e. most-affected). The boxplots on the right depict the FSHD biopsies included based on this classification. The two replicate FSHD samples sequenced in the two major sequence batches are labeled and highlighted in color. (**c**) Receiver Operator Characteristic curve for FSHD versus control biopsy classification with either DUX4 or PAX7 signature expression. AUC; area under curve. p-values depict the results of a Student’s t-test (excluding the replicate samples). p-values: ns = not significant, *p < 0.05, **p < 0.01, ***p < 0.001, ****p < 0.0001. All data plots are generated in R (v4.0.3, www.R-project.org) using the packages *gplots* (v3.1.1), *ggplot2* (v3.3.3) and *pROC* (v1.17.0.1). Figure and panel layout was further adapted in Adobe Illustrator CC 2018 (www.adobe.com).

We performed RNA sequencing on all muscle biopsies and evaluated DUX4 and PAX7 signature expression. Low expression levels and the sporadic nature of DUX4 target gene expression challenges the detection of DUX4 signature in global FSHD-associated differential expression analyses. We therefore used the cumulative expression of 57 known DUX4 target genes to study DUX4 activity in individual biopsy samples^23^. As expected, DUX4 signature expression was increased in FSHD compared to control biopsies (Table 1 and Fig. 2a). In control biopsies, the highest detected cumulative normalized read count for DUX4 signature was 13.7 (average 6.44, range 1.2–13.7), whereas the FSHD biopsies showed a wide range of DUX4 signature expression levels, with 19/39 (48.7%) biopsies showing a cumulative normalized read count > 20 (labeled DUX4-positive (DUX4^POS^); average 169.5, range 20.3–807.8). This increased cumulative read count was due to both a generally increased expression level per DUX4 target gene and an increased number of expressed DUX4 target genes per sample (Supplementary Fig. S2, Supplementary Table S3). The remaining 20/39 (51.3%) FSHD biopsies showed a DUX4 signature level similar to the controls (average cumulative normalized read count of 5.4, range 0–15.4 reads) and were labeled DUX4-negative (DUX4^NEG^) (Fig. 2a and Supplementary Fig. S2). Two DUX4^POS^ FSHD biopsies that served as technical replicates in the sample preparation and analysis of the control biopsies (see Methods) were classified DUX4^POS^ in both replicate analyses, indicating that the low level of DUX4 signature expression in the control batch was not due to detection bias (Fig. 2a and Supplementary Fig. S2). The technical replicates were excluded in all further analyses.

The PAX7 score is known to be reduced in FSHD compared to control muscle biopsies and myocytes^26,42^. Though, despite the detection of a reduced PAX7 score in some FSHD samples, differences were not significant in our sample set (Table 1 and Supplementary Table S2, Fig. 2b and Supplementary Fig. S2). One explanation could be that a reduced PAX7 score, like the DUX4 signature, represents a sporadic expression pattern in FSHD. Indeed, both DUX4 and PAX7 signatures showed only low-moderate FSHD classifier performance in Receiver Operator Characteristic (ROC) analysis (Fig. 2c), indicating that without additional biopsy selection criteria both signatures can only be detected in a subset of FSHD muscle biopsies. As a classification threshold for PAX7-affected versus non-affected biopsies could not be defined based on the scores in control biopsies, for further subgroup comparisons we classified the ten FSHD samples with the highest PAX7 score as PAX7^HIGH^ (i.e. likely non-affected) and the ten FSHD samples with lowest PAX7 scores as PAX7^LOW^ (i.e. most-affected) (Fig. 2b).

### Relations between metadata

**Figure 3:**
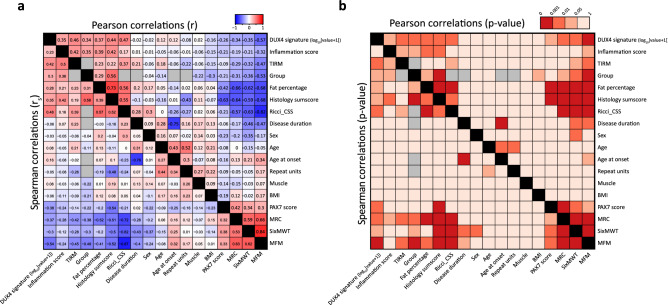
Linear correlation analysis between al metadata in this study’s dataset. (**a**) Pearson correlation scores r (top right values) and Spearman’s rank correlation scores r_s_ (bottom left) for all comparisons between the two molecular signatures (DUX4 and PAX7 signatures), the two imaging-based biomarkers (TIRM hyperintensity and fat fraction) and all metadata of our dataset (see also methods). (**b**) p-values for all correlations. Color-coding follows the correlation values and specific scores are indicated in each box. Grey boxes indicate that no linear correlation score could be calculated. Note that all results for non-muscle-specific metadata (i.e. age, age at onset, disease duration, group, CSS, D4Z4 repeat size, BMI, sex, 6-MWT and MFM) may be biased by duplicate samples for participants that donated a muscle biopsy from both the TA and VL muscle. The two duplicate VL muscle biopsies from participant FSHD-09 and FSHD-13 are also included in the data. This analysis indicates the strength of linear (for Pearson) and monotonic (for Spearman) correlations. For some metadata, other correlations may still apply. See also Supplementary Figs. S3 and S4 for detailed visualization of all quantitative correlations in this analysis. Pearson and Spearman’s rank correlation scores and p-values were calculated in R (v4.0.3, www.R-project.org) with the *cor.test* function of the *stats* package (v4.0.3). Data is plotted with the *heatmap.2* function of R package *gplots* (v3.1.1). Figure and panel layout was further adapted in Adobe Illustrator CC 2018 (www.adobe.com).

To evaluate for the presence of covariates or confounding factors in our muscle biopsy cohort, we performed a general correlation analysis between all metadata of our samples (Fig. 3a,b and Supplementary Fig. S3). As expected, functional outcome measures 6-MWT, MFM and MRC were strongly inter-related and 6-MWT and MFM furthermore negatively correlated with CSS and disease duration. MFM also correlated somewhat with age at onset, though our data suggests disease duration to have a bigger effect on functional outcome measures. Corresponding with its muscle-specific outcome within individual patients, MRC showed lower correlation scores with CSS, disease duration and age at onset. Functional outcome measures also correlated with muscle pathology, with increased histology sum scores associating with reduced functional outcome measures. Our groups did not differ in age, BMI and sex, and we did not find noticeable confounding effects for these factors with any of the disease-associated outcome measures (except for a moderate reduced 6-MWT distance in females). Although it has been described that the D4Z4 repeat size correlates with clinical severity and age at onset^43^, our dataset showed no significant (linear) correlation between these factors.

We next focused on molecular signatures expressions. Although PAX7 has been described to correlate with disease progression, we did not detect a significant difference in CSS in patients from the PAX7^HIGH^ versus PAX7^LOW^ group (though with PAX7^LOW^ samples trending to increased CSS, Supplementary Fig. S4). We did however detect a (linear) negative correlation between PAX7 scores and histology sum score, suggesting that a reduced PAX7 score is a better marker for progression of muscle pathology (Supplementary Fig. S4). We detected a limited correlation between histology sum score and DUX4 signature expression, but patients from the DUX4^POS^ biopsy group did show an increased average CSS, suggesting that DUX4 signature expression is correlated with disease progression, at least on a functional level (Supplementary Fig. S4). We did not detect a relation between the D4Z4 repeat size and DUX4 signature expression (Fig. 3a,b and Supplementary Fig. S3). Finally, as both DUX4 and PAX7 have been described to negatively regulate each other’s function^13^, one may expect both signatures to be negatively correlated in our dataset. However, although PAX7 scores were slightly reduced in DUX4^POS^ biopsies, we did not detect a clear correlation between both signatures, suggesting that both markers may represent a (partially) independent subset of FSHD biopsies (Supplementary Fig. S4).

For both imaging biomarkers, detection of either marker was significantly associated with increased CSS (Supplementary Fig. S4). TIRM hyperintensity is often associated with muscle inflammation and edema, and also in our dataset TIRM hyperintensity was significantly associated with signs of inflammation (Supplementary Fig. S4). Interestingly, TIRM hyperintensity did not associate with increased histopathology or disease duration, which may indicate that TIRM hyperintensity (and muscle inflammation) is dynamic and/or not limited to late stages of cellular pathology (Fig. 3, Supplementary Figs. S3 and S4). As expected, FSHD biopsies with increased fat fraction showed increased histopathology scores (Supplementary Fig. S4). Quantitative correlation analyses showed that whereas the histology sum score had a moderate quantitative correlation with fat fraction, CSS was not linearly correlated with fat fraction, with only samples from participants with a high CSS (≥ 6) showing increased fat fraction (Supplementary Fig. S4). This corresponds with previous reports that once fatty infiltration starts the fat fraction may rapidly increase and disease may quickly progress^31^.

FSHD is marked by heterogeneity in muscle involvement. Patients often present asymmetric muscle weakness and muscle involvement is not always the same during disease progression. Our dataset contained VL and TA muscle biopsies which are differently involved in FSHD. We therefore compared the muscle-specific measures (histology sum score, inflammation score, fat percentage, TIRM hyperintensity and MRC) in both muscle types individually and found similar trends between controls and FSHD biopsies in both (Supplementary Fig. S5, see also Supplementary Table S2 for all muscle-specific results for al comparisons in Table 1). However, the VL muscle seemed less fatty infiltrated and showed slightly better MRC scores, suggesting that the VL muscles were on average slightly less affected. We checked if this influenced the two transcriptome signatures, but did not see a clear difference in the pattern between control and FSHD muscle biopsies (Supplementary Fig. S5 and Supplementary Table S2). When analyzing the nine paired (VL–TA) FSHD muscle biopsy sets we observed a trend of increased DUX4 signature expression and reduced PAX7 score in the TA muscle, which corresponds with the TA muscle being often more affected than the VL muscle, though this trend did not reach statistical significance (Supplementary Fig. S5).

### TIRM hyperintensity and fat fraction as additive biomarkers for FSHD signature expression

To determine if imaging-based biomarkers can increase the detection of regions with disease signature expression, we analyzed the correlation between TIRM hyperintensity or increased fat fraction and both transcriptome signature expressions in our biopsies.

**Figure 4:**
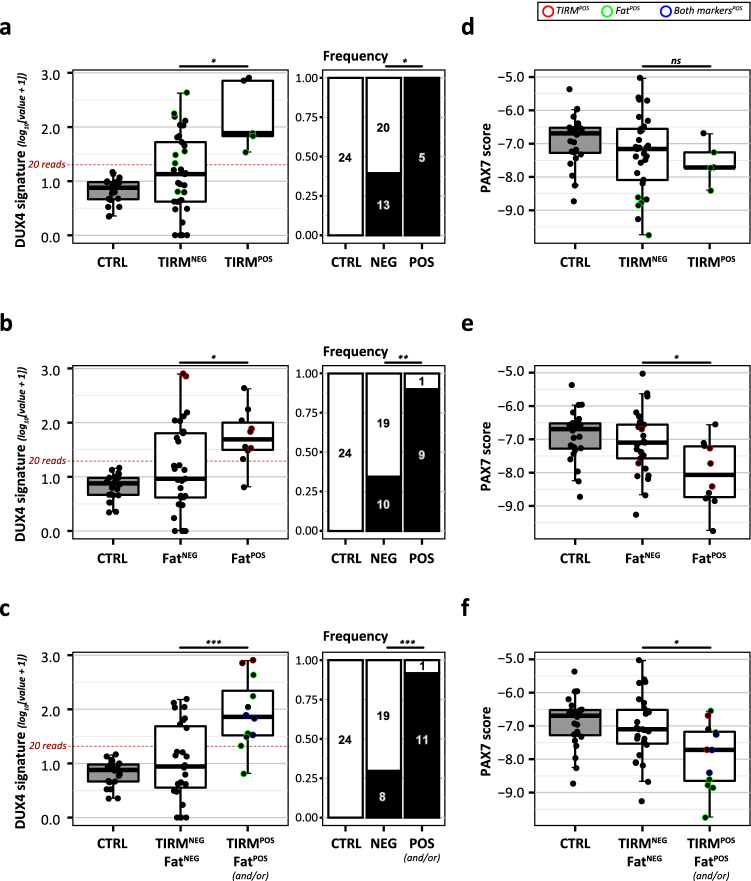
Correlations between DUX4 and PAX7 signature expression and TIRM hyperintensity and fat fraction. (**a**–**c**) DUX4 signature expression levels in control biopsies and FSHD biopsies separated based on imaging biomarker classifications, with in (**a**) TIRM hyperintensity, in (**b**) Fat fraction (Fat^POS^: > 15% Fat), an in (**c**) the combined biomarker (TIRM^POS^ and/or Fat^POS^). The threshold criterium for DUX4^POS^ biopsy selection (cumulative normalized read count > 20) is marked with a red dashed line. The stacked bar plots on the right depict the relative frequency of DUX4^POS^ biopsy classification in each subgroup. The numbers indicate the true frequencies. (**d**–**f**) PAX7 scores in control biopsies and FSHD biopsies separated based on imaging biomarker classifications, with in (**d**) TIRM hyperintensity, in (**e**) Fat fraction (Fat^POS^: > 15% Fat), an in (**f**) the combined biomarker (TIRM^POS^ and/or Fat^POS^). All p-values for the quantitative comparison of signature expression (boxplots) depict the results of a Student’s t-test comparing imaging biomarker-positive versus imaging biomarker-negative FSHD muscle biopsies respectively. For the frequency plots, the result of a Fisher’s exact test is depicted. For reference, in (**a**, **d**) Fat^POS^ biopsies are highlighted (green), in (**b**, **e**) TIRM^POS^ biopsies are highlighted (red) and in (**c**, **f**) both TIRM^POS^ (red), Fat^POS^ (green) and TIRM^POS^/Fat^POS^ (blue) biopsies are highlighted. p-values: ns = not significant, *p-value < 0.05, **p-value < 0.01, ***p-value < 0.001, ****p-value < 0.0001. All data plots are generated in R (v4.0.3, www.R-project.org) using the packages *gplots* (v.3.1.1) and *ggplot2* (v3.3.3). Figure and panel layout was further adapted in Adobe Illustrator CC 2018 (www.adobe.com).

Confirming previous findings, TIRM hyperintensity was associated with increased levels of DUX4 signature expression and all 5/5 (100%) TIRM^POS^ muscle biopsies were DUX4^POS^, with 2/5 TIRM^POS^ biopsies having the highest level of DUX4 signature expression of all biopsies (Fig. 4a). However, we also detected increased DUX4 signature expression in 13/33 (39%) TIRM^NEG^ FSHD muscle biopsies, indicating that whereas TIRM hyperintensity had a high specificity for DUX4^POS^ biopsies, its predictive power had limited sensitivity [5/19 (26%) DUX4^POS^ biopsies were TIRM^POS^]. The PAX7 score was not significantly reduced in TIRM^POS^ biopsies (Fig. 4d), indicating that TIRM hyperintensity in our cohort could not predict a reduced PAX7 score.

Fat fraction was also associated with DUX4 signature expression, with DUX4 signature levels being increased in Fat^POS^ FSHD biopsies and being significantly more often detected in Fat^POS^ [9/10 (90%)] compared to Fat^NEG^ FSHD biopsies [11/29 (40%)] (Fig. 4b). In our cohort fat infiltration could predict DUX4 signature expression with slightly higher sensitivity [9/19 (47.4%)] than TIRM hyperintensity. As only 3/5 TIRM^POS^ biopsies also had increased fat infiltration, inclusion of both fat fraction and TIRM hyperintensity as a biomarker for DUX4 signature expression, resulted in the highest sensitivity for DUX4^POS^ biopsies [11/19 (57.9%) DUX4^POS^ biopsies, Fig. 4c] without substantially reducing specificity [11/12 (91.6%) biomarker-positive FSHD biopsies are DUX4^POS^]. Increased fat fraction was, unlike TIRM, also associated with reduced PAX7 scores (Fig. 4e). Therefore, including fat fraction as a biomarker increased the detection rate of biopsies with either FSHD-associated signatures in our cohort (Fig. 4c,f).

DUX4 signature expression was also detected in 8/27 (29.6%) biopsies without imaging-based abnormalities (TIRM^NEG^ and Fat^NEG^). Interestingly, the participants from which these eight biopsies originated had mildly increased CSS scores as compared to DUX4^NEG^ muscle biopsies, (Supplemental Fig. S6) suggesting that these DUX4^POS^ biopsies show early signs of disease development possibly prior to (or without) signs of MRI-based biomarker abnormalities.

In addition, our cohort included two paired TIRM^POS^/TIRM^NEG^ biopsies from two adjacent regions of the same muscle (see Supplementary Table S1 for sample information). These paired biopsies can function as a well-controlled comparison of the effect of TIRM hyperintensity either in a Fat^NEG^ muscle context (i.e. possibly early stage disease progression, FSHD-13) or a Fat^POS^ muscle context (i.e. possibly late stage disease progression, FSHD-09). Although the sample size inhibited us from statistical analyses, we found that DUX4 signature levels were strongest increased in TIRM^POS^ biopsies when fat infiltration has not occurred (yet). Once fat infiltration has occurred, DUX4 signature levels may not increase as strongly upon signs of inflammation (Supplemental Fig. S6). This conclusion is strengthened by the cross-sectional comparison of all other biopsies and may indicate that fat infiltration (although correlating with increased DUX4 levels itself) may result in slightly lower detected levels of DUX4 signature. Part of this could be due to reduced muscle cell content in these biopsies, as correcting for fat fraction considerably increased DUX4 signature scores in some Fat^POS^ biopsies (Supplemental Fig. S7). Yet, a large variation in DUX4 signature expression remains even after fat correction and correcting for fat fraction did not increase the detection frequency of DUX4^POS^ biopsies in our muscle biopsy cohort. This indicates that myogenic content may only partially explain the heterogeneity in detected DUX4 signature expression. Also, correcting for myogenic content based on general myogenic marker gene expression (i.e. MYOD1 and MYOG), indicated that overall muscle cell content was not strongly affecting DUX4 signature detection (DUX4^POS^ classification) and only mildly affected signature levels in our cohort. Interestingly, correction based on specific fiber type markers indicated a positive correlation between DUX4 signature expression and regenerating myofibers (i.e. MYH3 and to a lesser extent MYH8), corresponding with the described increased signs of muscle regeneration in FSHD-affected muscle (Supplemental Fig. S7)^44^.

Opposite to DUX4 signature expression, PAX7 scores were strongest reduced in Fat^POS^ biopsies that lacked TIRM hyperintensity. As for the DUX4 signature, increased fat fraction may partially explain the reduced PAX7 score in some biopsies, as PAX7 scores were negatively correlated with increased fat fractions (Supplemental Fig. S7). In addition, based on myogenic marker gene expressions there may be a (limited) correlation between muscle cell content and PAX7 scores (Supplemental Fig. S7). Though, due to the fact that the PAX7 score is a t-statistic of upregulated and down-regulated PAX7 target genes and we do not know the theoretical t-statistic for these PAX7 target genes in fat and/or non-muscle cell types, we were unable to determine if the reduced myogenic content was fully responsible for the reduction in PAX7 score.

### Involvement of infiltrating non-muscle cells in FSHD

**Figure 5:**
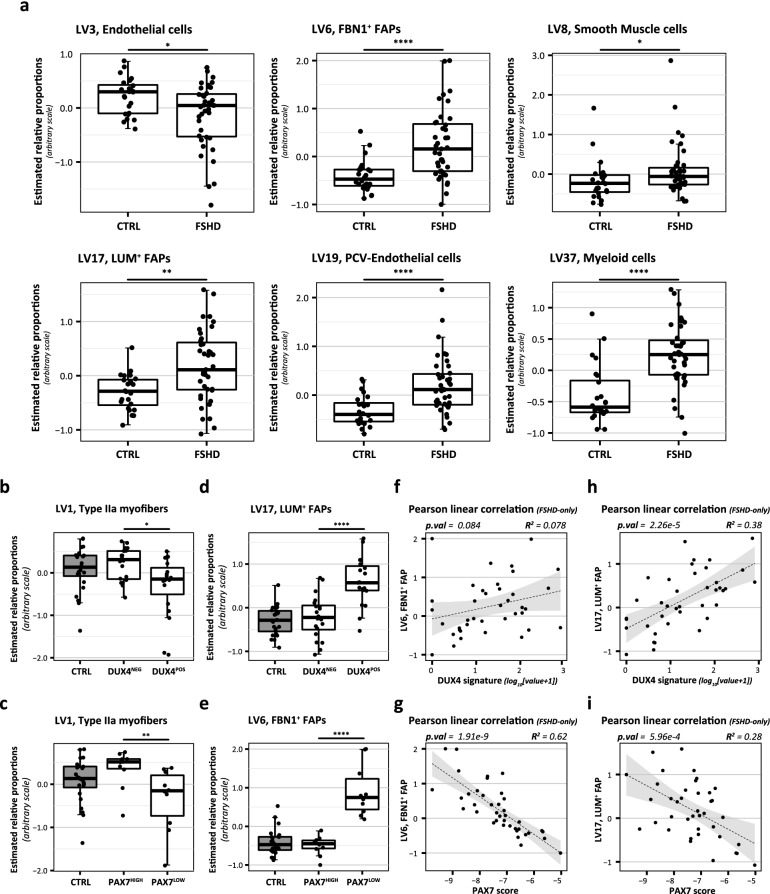
Significant non-muscle cell type contributions in FSHD, and their correlation with DUX4 and PAX7 signature expression. (**a**) Estimated relative contributions for all muscle and non-muscle cell types that show significantly different contributions in FSHD versus control muscle biopsies. See Supplemental Fig. S8 for the results of all (significant and non-significant) identified cell types. Results are based on the RNA deconvolution analysis (see “Methods” for details on PLIER analysis), based on cell types previously identified by Rubenstein AB et al.^45^ (in healthy human muscle biopsies). LV: latent vector best representing the respective cell type signature noted behind the LV number. FBN1 + FAPs: Fibrilin-1 positive fibro-adipogenic progenitors. LUM + FAPs: lumican-positive fibro-adipogenic progenitors. PCV-Endothelial cells: post-capillary venules endothelial cells. (**b**, **c**) Estimated relative contribution of the Type IIa myofiber content in controls and FSHD muscle biopsies showing a reduction in Type IIa myofiber content in both DUX4^POS^ versus DUX4^NEG^ FSHD biopsies (**b**) and in PAX7^LOW^ versus PAX7^HIGH^ FSHD biopsies (**c**). (**d**, **e**) Estimated relative contribution of the LUM^+^ FAP subtype in controls and DUX4^POS^ versus DUX4^NEG^ FSHD muscle biopsies (**d**) and FBN1^+^ FAP subtype in controls and PAX7^LOW^ versus PAX7^HIGH^ FSHD muscle biopsies (**e**). p-values in (**b**–**e**) depict the results of Mann–Whitney U tests. (**f**–**i)** Linear quantitative correlation analysis for both molecular signatures [DUX4 signature (**f**, **h**) and PAX7 score (**g**, **i**)] with each FAP subtype [FBN^+^ FAPs (**f**, **g**) LUM^+^ FAPs (**h**, **i**)], showing the strongest correlation of each molecular signature with a distinct FAP subtype. For quantitative correlations, only FSHD samples were included. Grey shadings indicate the 95%-confidence interval for the linear regression line. p-values and R^2^ values depict the result of a Pearson correlation. p-values: ns = not significant, *p < 0.05, **p < 0.01, ***p < 0.001, ****p < 0.0001. All data plots are generated in R (v4.0.3, www.R-project.org) using the packages *gplots* (v3.1.1), *ggplot2* (v3.3.3) and *ggpubr* (v0.4.0). Figure and panel layout was further adapted in Adobe Illustrator CC 2018 (www.adobe.com).

An important aspect of FSHD pathogenesis is the presence of muscle inflammation and the progressive muscle wasting, muscle fibrosis and fat infiltration. Skeletal muscle is composed of a complex mixture of muscle and non-muscle cell types, and the specific contribution of each cell type will affect the transcriptome. We performed an RNA deconvolution analysis to estimate the relative contribution of 12 different cell types present in healthy human muscles in vivo (see “Methods” for description of cell types)^45^. As expected, FSHD muscle biopsies had increased relative myeloid cell contributions, corresponding with muscle inflammation (Fig. 5a and Supplementary Fig. S8 for all cell type results). In addition, endothelial cell contributions were reduced in FSHD muscle biopsies, while post-capillary venule endothelial cells (PVC-endothelial) and smooth muscle cell signatures were increased. This may be a reflection of the complex tissue changes that occur during disease progression as the earliest RNA microarray studies in FSHD already identified a transcriptional dysregulation of vascular smooth muscle or endothelial cell genes^46,47^. Finally, we observed an increase of both defined fibro-adipogenic progenitor cell types (FAPs), which corresponds with the role for FAPs in response to muscle inflammation, regeneration and repair^48^. Of note, FAPs have been described to be increased after DUX4 expression in an inducible DUX4 mouse model, and FAPs may play a role in muscle fibrosis and fat replacement in FSHD^49^.

To test if any of the non-muscle cell types may affect FSHD signature expression we compared their contributions in signature-positive versus signature-negative muscle biopsy samples. DUX4^POS^ muscle biopsies followed a similar pattern of increased myeloid cells, increased FAPs and reduced endothelial cell contribution, though no association with increased smooth muscle cells and PVC-endothelial cells was found (Supplementary Figs. S9 and S11 for quantitative correlations). PAX7^LOW^ samples also followed the same pattern, but were associated with an increase in smooth muscle cells and PVC-endothelial cells (non-significant trend for the latter) and showed increased pericyte contribution (Supplementary Figs. S10 and S11 for quantitative correlations). Interestingly, although not detected based on individual fiber type marker gene analysis (Supplemental Fig. S7), both FSHD transcriptome signatures were associated with a reduction in Type IIa myofiber contribution (Fig. 5b,c and Supplementary Figs. S9, S10 and S11), which corresponds with previous reports on increased susceptibility of Type II fibers in FSHD and increased levels of Type I fibers in affected tissue^50^.

Since an earlier study described a possible direct role for infiltrating immune cells in FSHD signature expression, with a number of DUX4-induced genes being expressed by immortalized FSHD lymphoblastoid cell lines^51^, we evaluated the involvement of this cell type by analyzing the FSHD lymphoblast score defined in this study. As none of the 57 DUX4-target genes used in our study to calculate the DUX4 signature was included in the FSHD lymphoblast score, we do not expect this cell population to affect our DUX4 signature detection. Also, FSHD lymphoblast scores were not different between FSHD and control muscle biopsies (or in DUX4^NEG^ versus DUX4^POS^ biopsies) (Supplementary Fig S9), excluding a direct role for lymphoblasts in our detected FSHD transcriptome signatures.

Finally, we detected a linear correlation for both FSHD signatures with opposite FAP subtypes, with the PAX7 score showing a strong negative correlation with fibrilin-1 positive (FBN1^+^) FAPs, and DUX4 signature expression showing a stronger correlation with lumican-positive (LUM^+^) FAPs, respectively (Fig. 5d–i).

## Discussion

This study investigated the FSHD-associated DUX4 and PAX7 signature expression in 39 FSHD and 24 control biopsies (from 28 FSHD patients and 12 healthy individuals). This study tested their correlation with a variety of disease-associated factors, including CSS, disease duration, D4Z4 repeat size, muscle pathology scorings and functional outcome measures. In addition, this study analyzed the predictive power of two imaging-based biomarkers—Turbo Inversion Recovery Magnitude (TIRM) hyperintensity and fat fraction—as non-invasive biomarkers for FSHD signature detection in clinical trials. Furthermore, this study investigated the role of infiltrating non-muscle cell types in FSHD signature expression and detected distinct correlations between non-muscle cell contributions and both transcriptome signatures, including potentially distinct involvements for two fibro-adipogenic progenitor subtypes in FSHD.

The patient cohort included in this study represented a heterogeneous set of participants, varying in CSS, disease durations, age at onset and D4Z4 repeat sizes. We believe this to be a fair representation of the FSHD population, which also shows a wide degree of heterogeneity between patients. In addition, different from previous studies, in our study muscle selection was not MRI-informed, resulting in a representative cross-section of FSHD muscles as would be encountered in a clinical trial without prior imaging-based selection. The frequency of TIRM hyperintensity and the fat fractions detected in our cohort are consistent with previous reports for VL and TA muscles. Analyzing transcriptome signatures and imaging biomarkers in this heterogenous population will provide insights into the heterogeneity of both transcriptome signatures and the predictive power of both biomarkers in the clinic. Our metadata analysis showed no strong confounding effects of any non-disease-associated factors. In addition, no considerable difference was detected between both muscle types. Transcriptome signatures did not show strong correlations with repeat size, disease duration or age at onset but correlated with CSS and with both general and muscle-specific functional outcome measures.

As expected, TIRM hyperintensity was associated with inflammation and was not restricted to late stages of FSHD muscle pathology (based on histology sum scores and fat fraction). Fat fraction increased with increasing histopathology and its positive but non-linear correlation with CSS indicates that fat infiltration is associated with late stages of disease progression corresponding with the described accelerated disease progression once fat infiltration has started^18^.

Our findings solidify the value of TIRM as a biomarker for FSHD disease activity, and validate its use in preselecting muscle with an increased probability of expressing the DUX4 signature. However, TIRM hyperintensity has limited applicability as an FSHD biomarker in clinical trials, as the frequency of TIRM hyperintensity is relatively low (4–12% of lower limb muscles^33,35^), limiting participant selection and possibly excluding participants due to negative TIRM signals. Indeed, 13/33 (39.4%) TIRM^NEG^ muscle biopsies in our cohort also showed increased DUX4 signature expression (representing 13/19 (68%) DUX4^POS^ biopsies) and hence would be misclassified if TIRM-hyperintensity were used as the only biomarker for disease activity. Furthermore, not all TIRM hyperintense muscles show progressive fatty infiltration, and some muscles may progress in fat fraction without prior TIRM signal changes on muscle MRI^28,33^. Additional non-invasive biomarkers are therefore needed. We detected that increased fat fraction was associated with both DUX4 signature expression and reduced PAX7 scores. With elevated fat fraction being present in ~ 50% of lower limb muscles, fat fraction may therefore serve as an additional biomarker to identify muscles at risk for elevated DUX4 and/or reduced PAX7 signature expression. It is, however, important to note that caution is required for factors that may influence fatty infiltration in muscle independent from disease as this may lead to false positive selection of some muscles. The one Fat^POS^/DUX4^NEG^ biopsy in our sample set had a fat fraction of 28%, which was obtained from a female FSHD participant with a BMI of 34. A similar fat fraction of 26% was also observed in one female control participant with a BMI of 34. Hence, the increased fat percentage in this muscle may be attributable to obesity.

To date, there remains ongoing debate on the utility of proposed molecular (transcriptome) biomarkers in FSHD. With the causal role of DUX4 in FSHD pathophysiology, the DUX4 target gene signature is a well-acknowledged biomarker. However, its use may be limited by the spatio-temporally restricted nature of the DUX4 signature and the PAX7 score was suggested to be a more robust biomarker^26,42^. Though, the relevance and interpretation of the PAX7 score has been challenged because PAX7 is a developmental transcription factor that is not expressed in differentiated myocytes and DUX4 and PAX7 are not co-expressed^52,53^. In this study, we conducted an impartial analysis of both signatures in an independent muscle biopsy cohort to gain a better understanding of their spatio-temporal nature and their potential utility as FSHD biomarker. Altogether we found that both signatures display a sporadic expression pattern in FSHD-affected biopsies, each possibly correlating with different stages in disease activity and/or progression. We detected strongest DUX4 signature expression in muscle with TIRM hyperintensity that do not show increased fat fraction (yet). Moreover, DUX4 signature expression was detected in eight muscles that did not show signs of pathology on MRI. As these patients showed mildly increased CSS, these findings may highlight DUX4’s correlation with early stages of disease progression corresponding with its causal role in FSHD. In contrast, the observed correlation between reduced PAX7 scores and increasing muscle pathology may define a role for the PAX7 score as a biomarker for later stages of disease, after muscle wasting and fat replacement are initiated. This is in line with a recent study describing the PAX7 signature to be correlated with disease pathology and progression^26,27^. Of note, as we find no clear (negative) correlation between the DUX4 signature and PAX7 score, the PAX7 score may be an indirect biomarker, with the PAX7 score correlating with downstream induced muscle pathology independent of active DUX4 signature expression.

Our PAX7 score findings are different from previous studies as we did not detect significant overall reduction in PAX7 score in FSHD-affected biopsies compared to controls and the PAX7 score had limited strength as general biomarker for FSHD. Differences in biopsy cohort composition in the different studies may explain some of the deviations between our data and earlier studies. For example, the previous study included different muscle types (with only 17% and 39% of the biopsies originating from the VL and TA muscle, respectively). Furthermore, the published study preselected muscles by MRI, resulting in higher percentages of biopsies with MRI abnormalities. Finally, in the previous study the majority of TIRM^POS^ biopsies also showed increased fat fraction, possibly confounding their detected correlation of PAX7 with TIRM hyperintensity.

Our analyses of two pairs of adjacent biopsies from within the same muscle highlight the high degree of variability in DUX4 signature expression even within the same muscle. Although DUX4 signature expression may be strongest when directly targeting TIRM^POS^ regions, TIRM hyperintensity at any position within the biopsied muscle might suffice as biomarker for DUX4^pos^ detection as the TIRM^NEG^/Fat^NEG^ paired sample could also be classified as DUX4^POS^. This may possibly explain why studies do not find a perfect association between clinical severity or imaging-based biomarkers and the DUX4 signature. Our data therefore strongly supports a focal and temporal model of disease activity, at least as defined by the DUX4 signature and suggest that focal disease activity might need to be considered in clinical trial design and data analysis.

Our RNA deconvolution analysis provides insights into the contribution of non-muscle cells in FSHD muscle biopsies, and their correlation with FSHD signature expressions. Although the identification of distinct contributions of non-muscle cells in FSHD, and distinct correlations with DUX4 signature expression and PAX7 score is an interesting finding, a full understanding of the meaning of these correlations will require further investigation. Whether these cell types play a role in FSHD signature expression, and whether this role is direct or indirect (e.g. by reducing the relative myofiber content) remains to be further investigated. Similarly, the causality of the positive correlation between DUX4 signature expression and muscle regeneration markers (i.e. MYH3 and MYH8) cannot be conclusively determined with our dataset. Although this correlation fits the model in which DUX4-induced toxicity leads to increased muscle regeneration in FSHD affected muscle, it remains unknown whether the increased proportions of regenerating myofibers also play an active role in the increased DUX4 signature expression (as suggested by previous regeneration studies in transgenic mouse models for FSHD^11^). The increase in PCV-endothelium, smooth muscle cells and pericytes was a surprising finding as this may suggest an increase in microvasculature in FSHD muscle whereas capillary density has previously been shown to be reduced in FSHD^54^. It is possible that the increased signature detection is due to the previously described transcriptional dysregulation of vascular smooth muscle or endothelial cell genes^46^ in which case our deconvolution analysis may interpret increased signature activity as increased relative cell contribution. Although future validation studies will be required to draw strong conclusions on the contribution of these cell types, such cell type contributions may help us to better understand the cause and consequences of FSHD-associated vasculopathy. Finally, identifying distinct correlations between two FAP subtypes and both FSHD transcriptome signatures may indicate different roles for both subtypes in FSHD. Although limited is known on the identity and specific function of these FAP subtypes, based on a recent study on FAP involvement in FSHD it is tempting to speculate that both subtypes may represent different pathological states, either facilitating muscle regeneration (in early stages of disease, DUX4 signature-associated) or muscle fibrosis, fat accumulation and atrophy (in late stage of disease, PAX7 score-associated)^49^.

## Materials and methods

### Participants

Genetically confirmed FSHD patients were recruited from the Radboud University Medical Center^35,55^. Healthy individuals without a history of neuromuscular disease were included as controls. All participants were aged ≥ 18 years and had no contra-indications for muscle MRI or muscle biopsy. Length and weight were measured to calculate body mass index (BMI)^56^. Muscle strength was graded with the Medical Research Council (MRC) scale^57^. Functional performance was assessed using the 6-min walk test (6-MWT) and Motor Function Measure (MFM), a 32-item scale which measures the functional abilities of a person affected with neuromuscular disease, expressed as a percentage of maximal functional performance^58,59^. FSHD disease severity was assessed using the 10-grade Ricci Clinical Severity Score (CSS) (using integer numbers scale, which is a doubling of the original CSS)^60^. Summary metrics for all included participants are provided in Table 1.

### Standard protocol approvals, registrations, and patient consents

The Medical Ethics Review Committee region Arnhem-Nijmegen approved this study. All experiments were performed in accordance with relevant guidelines and regulations. Informed consent was obtained from all individual participants included in the study.

### Muscle biopsy collection

VL biopsies were performed at about 1/3 of the distance between patella and anterior superior iliac spine (ASIS). All controls and 12 FSHD participants also contributed a second muscle biopsy of the TA muscle, which was taken at the point of maximum muscle bulk of the TA. Bergström needle biopsies were performed by an experienced physician taking routine antiseptic precautions^61^. Biopsies were performed under local anesthesia (lidocaine) and approximately 100 mg of tissue was collected per biopsy. A subset of FSHD VL muscle biopsies was performed with MRI-guidance by an intervention radiologist^62^. This allowed us to obtain samples from sites with focal TIRM hyperintensities. An overview of all collected muscle biopsies and associated metadata is provided in Supplementary Table S1. Muscle biopsies were snap-frozen in cooled isopentane (for histopathology analysis) or liquid nitrogen (for RNA-sequencing) and stored at − 80 °C.

### Immunohistochemistry

For histopathological analysis, frozen sections were stained with hematoxylin phloxine (Hphlox) staining. Stained sections were evaluated for variability in fiber size, extent of central nucleation, necrosis and regeneration, and interstitial fibrosis, which were graded manually as normal (0), mild (1), moderate (2) or severe (3). Severity scores of these 4 parameters were then summed to provide a cumulative histopathological sum score between 0 and 12^63^. All histopathology sum scores were assigned by an experienced neuropathologist in a blinded analysis. A minor increase in central nucleation < 3% was considered normal (0). Interstitial fibrosis in a focal area of the muscle biopsy was scored as 1, whereas more extensive interstitial fibrosis was scored as ≥ 2 depending on the severity of abnormalities. The presence of any necrosis and/or regeneration on Hphlox staining was considered abnormal and scored as ≥ 1 depending on the severity of abnormalities. The presence of any inflammatory infiltrate was considered abnormal and scored as ≥ 1 depending on the severity of abnormalities.

### Quantitative muscle MRI

#### MRI scanning protocols

TIRM sequences and transversal T1-weighted, multi-echo T2 images or Dixon sequences were acquired of the upper and lower leg of all except for two participants (Supplementary Table S1): upper leg MRI scanning was not possible in one control participant (CTRL-05_VL) due to claustrophobia, and TIRM images were not acquired in one FSHD participant (FSHD-10_TA).

MRI imaging of the upper and lower leg was performed according to previously described MRI protocols^35,64^. In 12/12 controls and 14/28 FSHD participants, transversal T1 weighted, multi-echo T2 images and Turbo Inversion Recovery sequences (TIRM) of the upper and lower leg were acquired on a 3 Tesla MRI system (Tim TRIO, Siemens, Erlangen, Germany) (see Supplementary Table S1 for included participants). Participants were placed in the scanner feet first supine using a spine array coil and two phased-array coils placed around the legs. Scout images were acquired in three orthogonal directions for accurate positioning of the MRI slices, centered on a fish-oil marker which was placed on the skin at the 1/3 of the distance between patella and ASIS. Eight transversal slices (FOV 175 × 175 mm^2^, thickness 4 mm, gap 6 mm, base resolution 256) were acquired with a T2 multi spin echo sequence (TR: 3000 ms, 16 equally spaced echo times 7.7–123.2 ms). Next, 23 transversal slices (thickness 4 mm, gap 0.4 mm) were obtained with a T1 turbo spin echo sequence (FOV 250 × 244.5 mm^2^, TR/TE 600 ms/13 ms, base resolution 448), and with a TIRM sequence (FOV 175 × 175 mm^2^, TR/TE/TI 4100 ms/41 ms/220 ms, base resolution 256). The same imaging protocol was used for both the upper and lower leg.

In 14/28 FSHD participants transverse Dixon and TIRM sequences of the upper and lower leg were acquired on the same 3 Tesla MRI system (see Supplementary Table S1 for included participants). Participants were placed in the scanner feet first supine. Scout images were made in 3 orthogonal directions to position imaging slices for subsequent scans. A transverse Dixon sequence was acquired around the upper and lower leg (field of view 271 × 435 mm, matrix size 200 × 320, repetition time 10 ms, echo time 2.45/3.675 ms, number of slices 144, slice thickness 5 mm, slice gap 0 mm, flip angle [FA] 3°). For the TIRM sequence, the inversion time was selected to null the fat signals (field of view 271 × 435, matrix size 160 × 256, repetition time 4,000 ms, echo time 40 ms, inversion time 220 ms, number of slices 72, slice thickness 5 mm, slice gap 5 mm, FA 150°).

#### Quantitative fat fraction analysis

The multi-echo T2 images or Dixon fat fraction maps were used to calculate fat fractions for all muscles, except for one control participant (CTRL-02_TA) because of imaging artefacts (Supplementary Table S1). Fat fraction analysis was performed on the image slice corresponding to 1/3 of the distance between patella and ASIS, i.e. the prospective muscle biopsy site. The fat fractions of the VL and TA was quantified by manually tracing the muscle outline of the muscle on the multi-echo T2 images or Dixon fat fraction maps. For MRI-guided muscle biopsies, fat fraction was determined in a ~ 1 cm circular area surrounding the MRI-guided biopsy site.

Based on previous studies in healthy controls, fat fraction of 15% was used as the cut-off point for normal (≤ 15%, Fat^NEG^) and abnormal (> 15% Fat^POS^) fatty infiltration^31,36,40,41^. Importantly, determination of fat fraction at the area surrounding the MRI-guided muscle biopsy did not result in a different classification of the muscle compared to determination of fat fraction of the entire muscle. Muscle fat fractions determined by Dixon were relatively low (all < 15% except for one participant with a muscle fat fraction of 25%). In our experience, quantitative analysis based on Dixon images overestimates fat fraction relative to analysis based on T2 images at low fat fractions. Hence, measurement using T2 would not have resulted in higher fat fractions in these muscles.

#### TIRM hyperintensity

TIRM images were scored by two members of the research team independently (SL, KM). TIRM positivity was a binary determination based on the T2 signal. Muscle biopsies acquired from TIRM hyperintense muscles or areas are denoted as TIRM^POS^, whereas muscle biopsies acquired from TIRM negative muscles or areas are TIRM^NEG^.

### RNA sequencing

#### Sample processing

For RNA isolations, a small piece of each muscle biopsy sample (~ 10 mg) was submerged into 700 μl Qiazol lysis reagent (Qiagen cat.nr 79306) directly from − 80 °C. Tissue was lysed using an ultra-turrax T25 homogenizer and RNA was isolated using the miRNeasy mini RNA isolation kit (Qiagen cat.nr 217004), according to manufacturer’s protocol (including a 30 min on-column DNAse I (Qiagen cat.nr 79254) treatment). RNA quality was checked on an Agilent BioAnalyzer RNA Nano 6000 chip (cat.nr 5067–1511) or Agilent Fragment Analyzer and all RNA samples had an RNA Integrity Number (RIN)/RNA Quality Number (RQN) ≥ 6.6 (with 53/65 samples ≥ 8). Technical replicate samples of two FSHD-affected biopsies (FSHD-02_VL and FSHD-13_VL) were included in the two major sequencing batches to exclude any bias in signature detection. For this, a new sample of the identical muscle biopsy was included in the RNA isolation, sequencing library preparation and sequencing of the second batch of control biopsies.

#### Sequencing and sequence analysis pipeline

For all samples except FSHD-09_VL1 and FSHD-09_VL2, polyA-tailed RNA sequence libraries were generated with the NEBNext Ultra (or Ultra II) Directional RNA Library Prep Kit for Illumina (NEB #E7420S/L and NEB #E7760S/L) according to manufacturer’s protocol. Image analysis, base calling, and quality check was performed with the NextSeq 500 RTA software (v2.4.11/v3.4.4, Illumina)^65^ and Bcl2fastq (v2.20, Illumina)^66^. PolyA-tailed RNA sample sequence libraries for samples FSHD-09_VL1 and FSHD-09_VL2 were generated separately. For these, polyadenylated transcripts were enriched using Oligo(dT) Dynabeads (Invitrogen, cat.no. 61005) and fragmented at 94 °C for 4 min. First strand synthesis was performed using random hexamers (Invitrogen, cat.no N8080127) and SuperScript III reverse transcriptase (Invitrogen, cat.no 18080044) according to the manufacturer’s protocol. Second strand synthesis was performed using uracil in place of thymine followed by Kapa HyperPrep kit library preparation (Roche, kk8504). Image analysis, base calling, and quality check was performed with the NextSeq 500 RTA software (v2.7.7, Illumina)^65^ and Bcl2fastq (v2.20, Illumina)^66^.

Reads were trimmed and quality filtered by TrimGalore (v0.4.5, cutadapt v1.16)^67^ using default parameters and mapped to Genome Reference Consortium Human Build 38 (GRCh38, Gencode release 28)^68^ using STAR aligner (v2.5.1b)^69^. A gene expression counts table was generated using HTSeq (v0.9.1, genome annotation hg38) ^70^. All biopsy samples were sequenced at an average sequencing depth of ~ 30 × 10^6^ reads (average raw read count: 29,450,900 reads; range 23,280,112–55,280,984 reads).

#### DUX4 signature analysis

DUX4 signature expression was determined by using the previously described 67 DUX4 target genes [of which 57 genes remained in the new genome build (GRCh38)]^23^. With the sporadic nature of DUX4 and DUX4 target expression, we used the cumulative normalized read count of all 57 target genes as DUX4 signature expression score. Data was first sequence depth-normalized following the median of ratios method implemented in DESeq2 R Package (v1.24.0, average normalized read count: 28,756,122; range 18,437,383–36,563,718)^71^. The expression levels of the individual DUX4 target genes used to calculate the DUX4 signature expression are listed in Supplementary Table S3. DUX4^POS^ biopsies were classified based on a cumulative normalized read count of > 20 target gene reads, whereas DUX4^NEG^ biopsies had a cumulative normalized read count of ≤ 20. For statistical analyses on the DUX4 signature expression levels, we log-transformed the signature score (log_10_[value + 1]) to fit a normal distribution (see also Statistics section).

#### PAX7 signature score

The PAX7 signature score was calculated as described previously and is defined as the *t*-statistic comparing the expression of induced and repressed PAX7 targets genes within the biopsy sample^26,42^. To ensure best comparison with literature, we log-transformed (log_10_(value + 1)) and quantile-normalized our data similar to described in the publication. The expression levels of the individual PAX7 target genes used to calculate the PAX7 score are listed in Supplementary Table S4.

### Statistics

All downstream analyses were further performed with R v4.0.3^72^. All plots are generated using the R packages *gplots* (v3.1.1), *ggplot2* (v3.3.3) and *ggpubr* (v0.4.0). The used statistical test is noted with every outcome and in the figure legends. The choice of statistical test is based on whether the data was normally distributed, which was tested by a Shapiro–Wilk test (*stats* package in R, v4.0.3, *shapiro.test()* function) and visual inspection of the data by quantile–quantile plots (*ggpubr* package in R, v0.4.0, *ggqqplot()* function): normally distributed data was analyzed with a Student’s t-test (*stats* package in R, v4.0.3, *t.test()* function); non-normally distributed data was analyzed with a Mann–Whitney *U* test (also known as a Wilcoxon rank-sum test, *stats* package in R, v4.0.3, *wilcox.test()* function); and classification data was analyzed with a Fisher’s exact test (*stats* package in R, v4.0.3, *fisher.test()* function). All tests are two-sided tests and Student’s t-tests are Welch-corrected for unequal variance. All p-values are indicated with the same asterisk labeling: ns = not significant, *p < 0.05, **p < 0.01, ***p < 0.001, ****p < 0.0001.

Receiver Operator Characteristic analyses for the DUX4 signature (log-transformed) and the PAX7 score were performed using the *pROC* package in R (v1.17.0.1).

Pearson’s correlations and Spearman’s rank correlations were calculated with the *stats* package in R (v4.0.3, *cor.test()* function). For individual Pearson linear correlations with disease-associated factors or fat fractions (Fig. 5 and Supplementary Figs. S4 and S6); only FSHD muscle biopsies were included to prevent bias from the many negative control biopsies in the dataset. For the complete overview of all relations in the metadata of our sample set (Fig. 3 and Supplementary Fig. S3); all samples were included. Supplementary Fig. S3 was generated with the *GGally* package in R (v2.1.1, *ggpairs()* function). Both Pearson’s correlation and Spearman’s rank correlation scores were provided to allow better comparison of all different data types. Pearson correlations indicate the strength of linear correlations only, and a low correlation score does not exclude the possibility for non-linear correlations. Spearman’s rank correlation does not assume linearity, but assesses monotonic relationships. Note that all results for non-muscle-specific metadata (i.e. age, age at onset, disease duration, group, CSS, D4Z4 repeat size, BMI, sex, 6-MWT and MFM) may be biased by duplicate samples for participants that donated a muscle biopsy from both the TA and VL muscle. The two duplicate VL muscle biopsies from participant FSHD-09 and FSHD-13 are also included in the data.

### RNA deconvolution analysis for cell type contributions

RNA deconvolution was performed using the Pathway-Level Information ExtractoR (PLIER) software (*PLIER* package in R, v0.99.0, default settings)^73^. Marker gene panels for all included cell types were based on the recent paper from Rubenstein et al.^45^. Eleven high-confidence latent vectors (AUC > 0.7, FDR < 0.05 and max per cell type = top 3) could be identified. This excluded satellite cells from our analysis as no representative LV for this cell type could be detected with high confidence. In addition, the B and T cells signature overlapped with *LV5*, *NK cells* and *LV37*, *Myeloid cells*, suggesting that contributions of these cell types cannot be discriminated. Finally, *LV22*, *Endothelial cells* was excluded from the analysis due to its overlap with other cells types and its low U coefficient as compared to *LV3*, *Endothelial cells*.

## Supplementary Information

Supplementary Table S1.

Supplementary Table S2.

Supplementary Table S3.

Supplementary Table S4.

Supplementary Figures.

## Data Availability

All processed transcriptomics data derived in this study and used to generate the DUX4 and PAX7 molecular expression signature scores are provided in the Supplemental data (Supplementary Table S3 and Supplementary Table S4, normalized read counts). All RNA-seq data (fastq files and processed read counts table along with their associated meta data) are deposited in the European Genome-Phenome Archive (EGA) under controlled access. Data is accessible through Dataset-ID EGAD00001008337 upon reasonable request.
