# Supplementary material for: Facioscapulohumeral dystrophy transcriptome signatures correlate with different stages of disease and are marked by different MRI biomarkers
Source: Sci Rep. 2022 Jan 26;12:1426. doi: 10.1038/s41598-022-04817-8 (PMC8791933; doi:10.1038/s41598-022-04817-8)
Supplement: Supplementary file 5 — Supplementary Figures. [file 41598_2022_4817_MOESM5_ESM.pdf]

Supplementary Figure S1

Facioscapulohumeral dystrophy transcriptome signatures correlate with different stages of disease and are marked by different MRI biomarkers

Van den Heuvel, A & Lassche, S et al

submission ID: 631a14da-1e22-4289-88d4-f5e7e5973f60

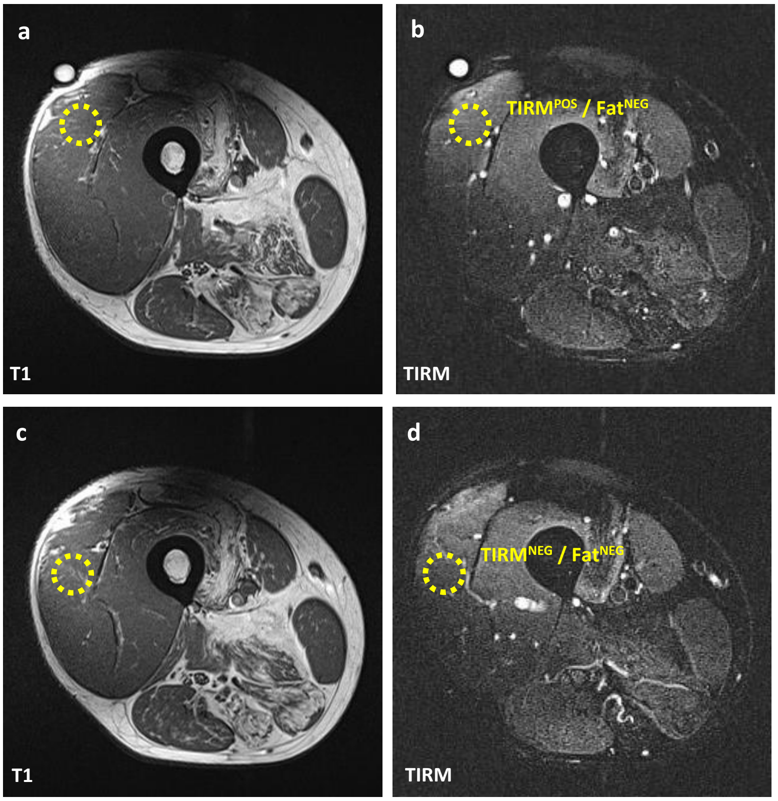

Supplementary Figure S1. Separate biopsies from TIRM^{POS} and TIRM^{NEG} areas within the same muscle.
a,c) Axial T1-weighted and **b,d)** TIRM images of the right upper leg of a 55-year-old FSHD patient (FSHD-13) showing mild fatty infiltration of the anterior part of the vastus lateralis and focal TIRM hyperintensity adjacent to this. In T1: Normal muscle is dark grey, fat infiltrated muscle is white. The MRI-guided biopsy sites are marked with the yellow circles. The Fat^{NEG}/TIRM^{POS} MRI-guided biopsy area is situated at the periphery of the TIRM hyperintense area. The Fat^{NEG}/TIRM^{NEG} MRI-guided biopsy site is located in a TIRM negative area. MRI scans were exported from Agfa IMPAX (https://global.agfahealthcare.com) and figure and panel layout was further adapted in Microsoft Powerpoint (www.microsoft.com).

Supplementary Figure S2

Facioscapulohumeral dystrophy transcriptome signatures correlate with different stages of disease and are marked by different MRI biomarkers

Van den Heuvel, A & Lassche, S et al

submission ID: 631a14da-1e22-4289-88d4-f5e7e5973f60

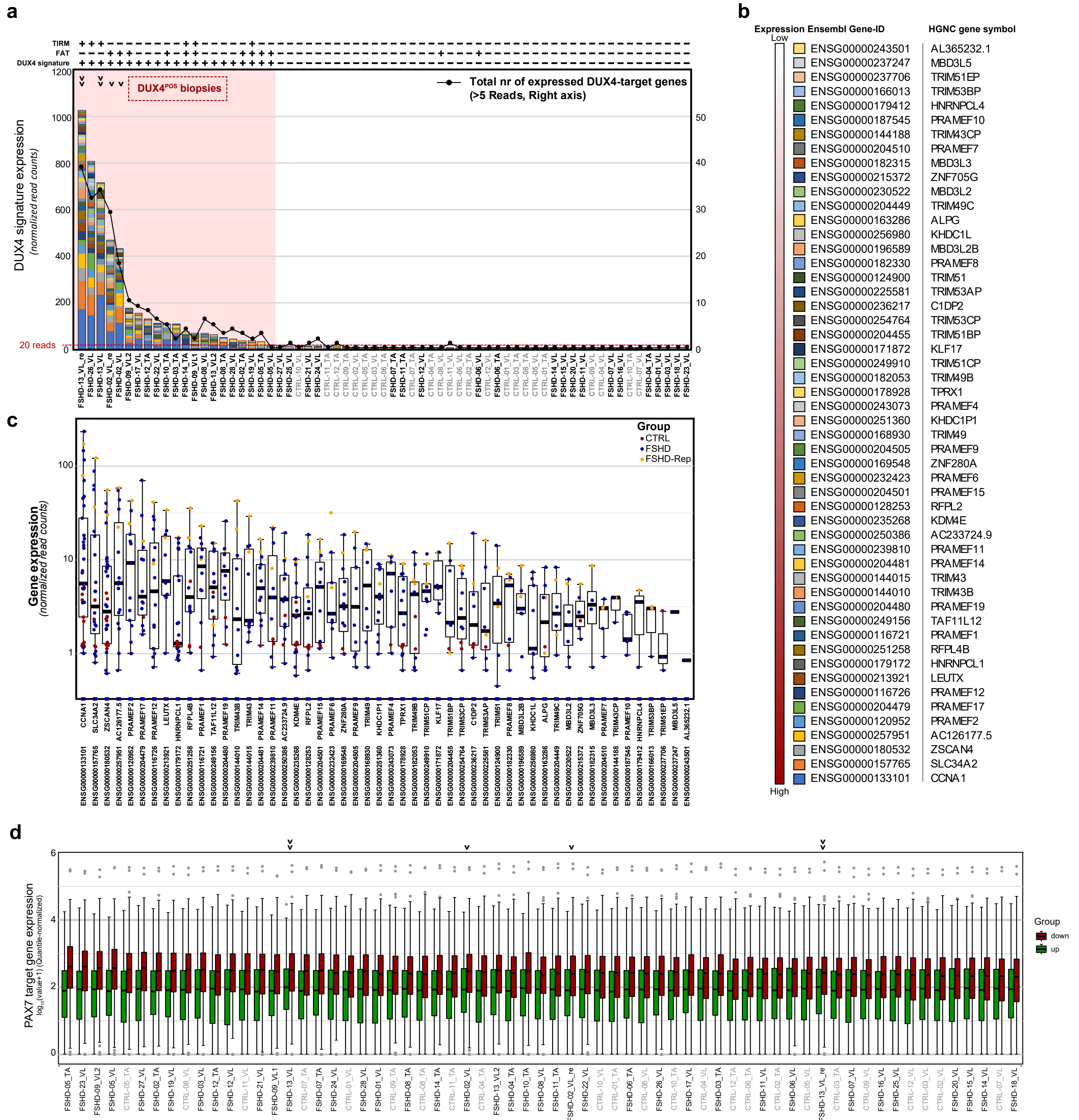

Supplementary Figure S2. DUX4 and PAX7 signature expression in individual muscle biopsies.

a) Stacked bar chart depicting the normalized read counts for all 57 individual DUX4 target genes of the DUX4 expression signature, per muscle biopsy. Samples are sorted from left to right by DUX4 signature expression level. FSHD samples are labeled in black, control samples in grey. Genes are sorted from bottom to top, based on the average expression level in all samples. The threshold criterium for DUX4^{POS} biopsy selection (normalized read count >20) is marked with a red dotted line. DUX4^{POS} biopsies are highlighted by the red block. The two replicates are marked (v) and ($\overset{v}{v}$). The line-graph depicts the total number of DUX4-target genes with a normalized read count >5 (using right axis). **b)** The legend for all 57 DUX4 target genes in the stacked bar chart in a). **c)** Box and dotplot depicting the individual normalized expression levels for each DUX4 target gene. Genes are sorted from left to right by average expression in all samples. Samples with value=0 are depicted at the base of the plot and were excluded from the boxplot. All boxplots depict the 25-75% quantile range, whiskers extend to respectively highest or lowest point with a maximum extension of 1.5x inter-quantile range. **d)** Boxplots depicting the individual normalized expression levels for all upregulated (green) or downregulated (red) PAX7 target genes used to calculate the PAX7 score, per muscle biopsy. Biopsies are sorted from left to right based on PAX7 score (lowest PAX7 score on the left). Samples with value=0 are depicted at the base of the plot and were excluded from the boxplot. All boxplots depict the 25-75% quantile range, whiskers extend to respectively highest or lowest point with a maximum extension of 1.5x inter-quantile range. Outliers are depicted in grey. The two replicates are marked (v) and ($\overset{v}{v}$). Panel a) is generated in Microsoft Excel (www.microsoft.com). Panel c) and d) are generated in R (v4.0.3, www.R-project.org) using the packages *gplots* (v3.1.1) and *ggplot2* (v3.3.3). Panel b) is generated based on the figure legend of panel a) in Microsoft Excel. Figures and panel layout was further adapted in Adobe Illustrator CC 2018 (www.adobe.com).

Supplementary Figure S3

Facioscapulohumeral dystrophy transcriptome signatures correlate with different stages of disease and are marked by different MRI biomarkers

Van den Heuvel, A & Lassche, S et al

submission ID: 631a14da-1e22-4289-88d4-f5e7e5973f60

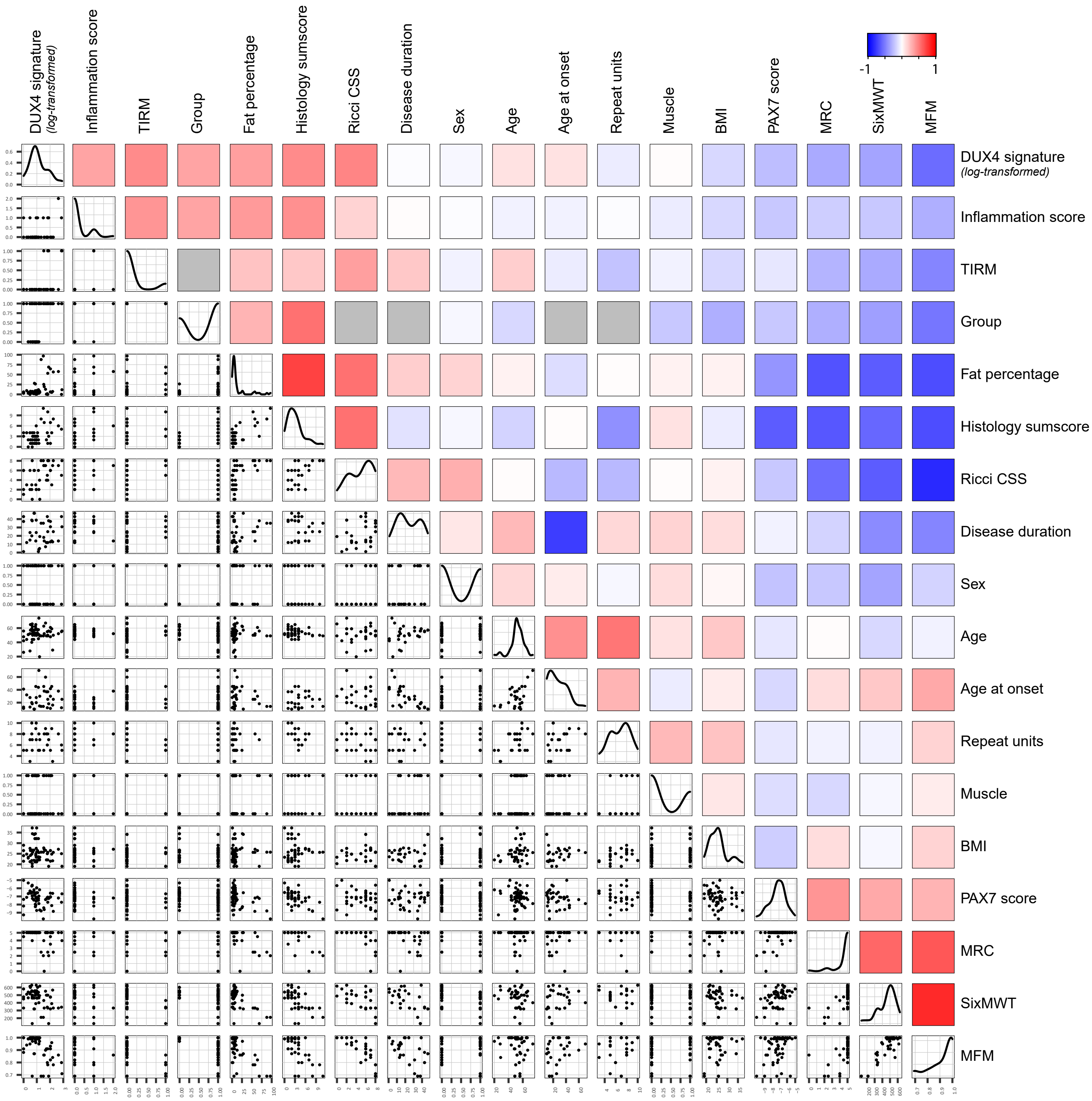

Supplementary Figure S3. Correlation plots for all metadata.

Quantitative correlation plots for all included metadata in our dataset. Color-coding represents the Pearson (linear) correlation scores as in Figure 3a. Grey boxes indicate that no linear correlation score could be calculated. Note that all results for non-muscle-specific metadata (i.e. age, age at onset, disease duration, group, CSS, D4Z4 repeat size, BMI, sex, 6-MWT and MFM) may be biased by duplicate samples for participants that donated a muscle biopsy from both the TA and VL muscle. The two duplicate VL muscle biopsies from participant FSHD-09 and FSHD-13 are also included in the data. Pearson correlation scores indicate strength of linear correlations, and a low correlation score does not exclude the possibility for non-linear correlations. As a better comparison for not-normally distributed factors, the Spearman correlation scores are included in Figure 3b. All data plots are generated in R (v4.0.3, www.R-project.org) using the package *GGally* (v2.1.1). The color-coding is based on the correlation values and is generated with the *heatmap.2* function of the *gplots* package (v3.1.1). Figure and panel layout was further adapted in Adobe illustrator CC 2018 (www.adobe.com).

Supplementary Figure S4

Facioscapulohumeral dystrophy transcriptome signatures correlate with different stages of disease and are marked by different MRI biomarkers

Van den Heuvel, A & Lassche, S et al

submission ID: 631a14da-1e22-4289-88d4-f5e7e5973f60

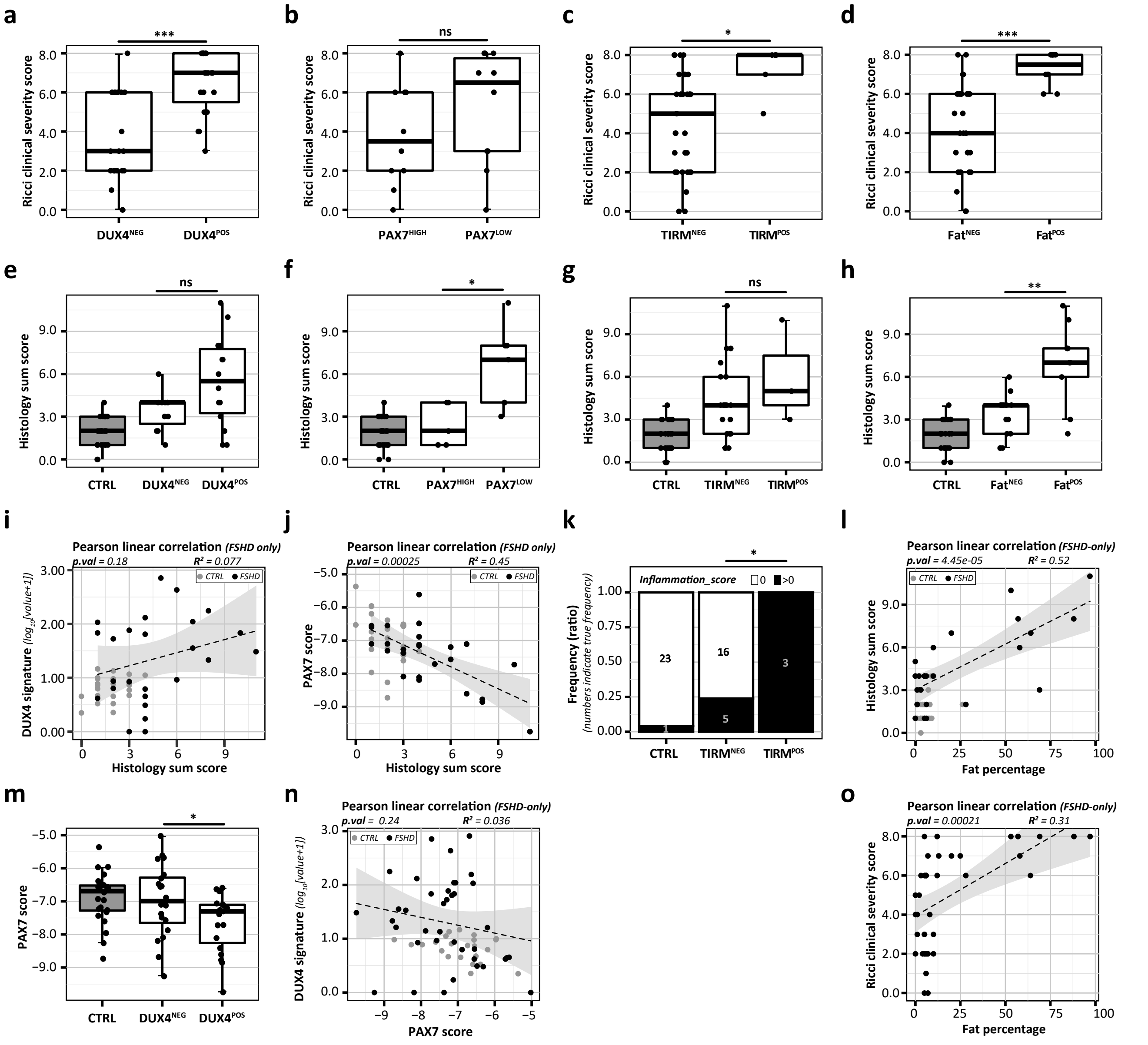

Supplementary Figure S4. Highlighted correlation plots for both molecular signatures and imaging-based biomarkers.

a-d) Analysis of CSS in both molecular signatures **(a, b)** and imaging-based biomarkers **(c, d)**. p-values depict the results of a Mann-Whitney U test. **e-h)** Analysis of histology sum scores in both molecular signatures **(e, f)** and imaging-based biopmarkers **(g, h)**. p-values depict the results of a Mann-Whitney U test on all FSHD-samples for which a histology sum score was determined (n = 25, see Supplementary Table 2). **i-j,l)** Linear correlation analysis for DUX4 signature expression **(i)**, PAX7 score **(j)** and fat fraction **(l)** with histology sum score. p-values and R^2 values depict the results of a Pearson correlation test on all FSHD-samples for which a histology sum score was determined (n = 25, see Supplementary Table S2). **k)** Detection frequency of inflammation in $TIRM^{POS}$ versus $TIRM^{NEG}$ FSHD muscle biopsies. p-value depicts the result of a Fisher's exact test comparing no inflammation (score = 0) versus signs of inflammation (score > 0). **m, n)** Analysis of the correlation between PAX7 score and DUX4 signature expression, with in **m)** showing a slight reduction in PAX7 score in $DUX4^{POS}$ versus $DUX4^{NEG}$ subgroups and in **n)** showing the absence of a linear correlation between the two signatures. p-values depict the results of a Students' t-test (in **m**) and a Pearson correlation (in **n**). **o)** Pearson correlation for fat fraction versus CSS. p-value and R^2 value depict the results of a Pearson correlation test on all FSHD-samples. For all linear regression plots; grey shading depicts the 95%-confidence interval of the linear regression line. p-values: ns = not significant, * = p < 0.05, ** = p < 0.01, *** = p < 0.001, **** = p < 0.0001. All data plots are generated in R (v4.0.3, www.R-project.org) using the packages *ggplot2* (v3.3.3) and *ggpubr* (v0.4.0). Figure and panel layout was further adapted in Adobe illustrator CC 2018 (www.adobe.com).

Supplementary Figure S5

Facioscapulohumeral dystrophy transcriptome signatures correlate with different stages of disease and are marked by different MRI biomarkers

Van den Heuvel, A & Lassche, S et al

submission ID: 631a14da-1e22-4289-88d4-f5e7e5973f60

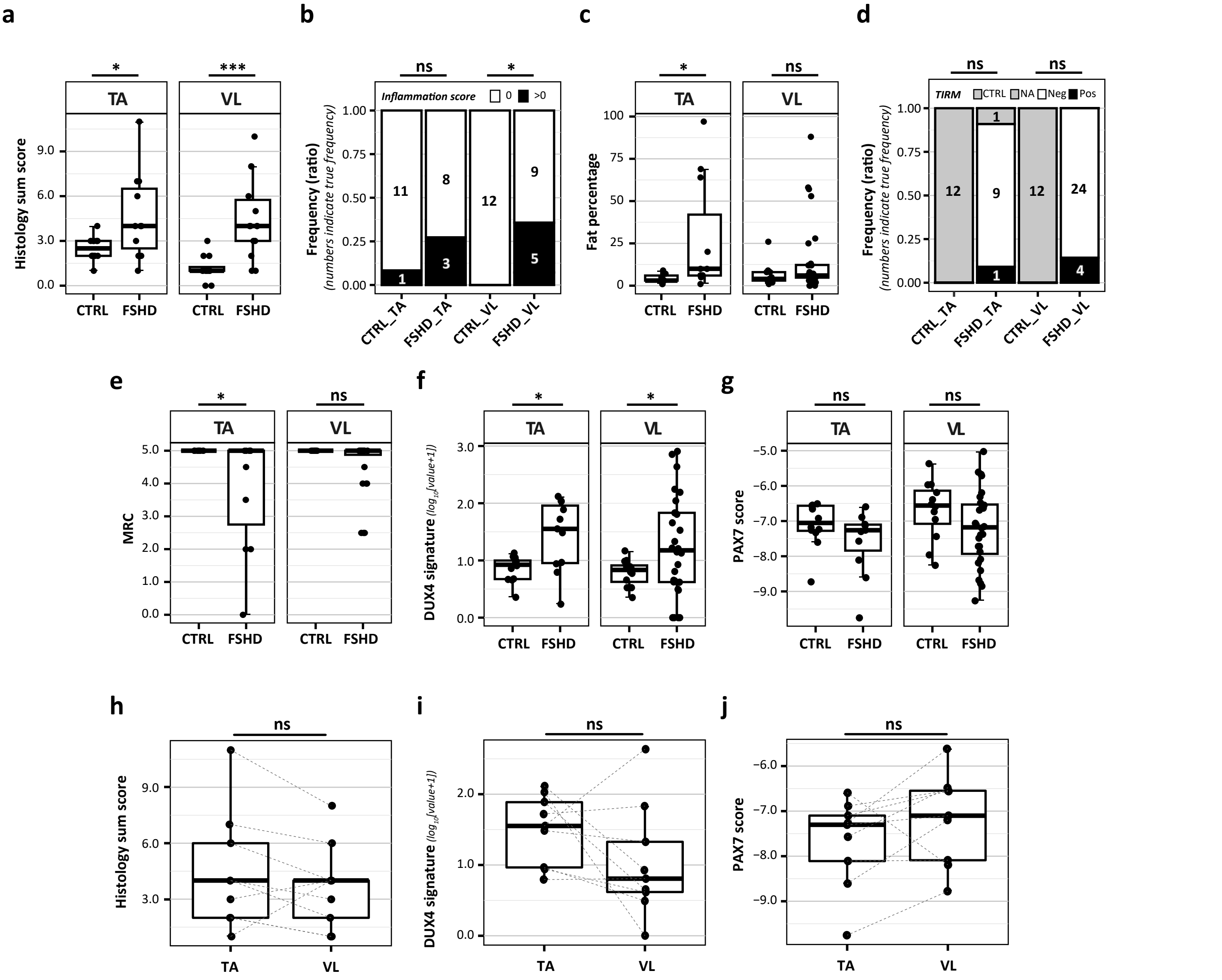

Supplementary Figures S5. Analysis for a muscle bias in the disease-associated outcome measures, muscle pathology and imaging and molecular biomarker detection.
a-b) Analysis of muscle pathology scores (histology sum scores **(a)** and inflammation scores **(b)**) in the individual muscle types, showing similar patterns between control and FSHD biopsies. p-values depict the results of a Mann-Whitney U test (in **a**) and a Fisher's exact test (in **b**). **c-d)** Analysis of imaging biomarkers (fat fraction **(c)** and TIRM hyperintensity **(d))** in the individual muscle types, showing similar patterns between control and FSHD biopsies, except for a slightly lower fat fraction in the VL muscle. p-values depict the results of a Mann-Whitney U test (in **c**) and a Fisher's exact test for no inflammation (score = 0) versus signs of inflammation (score > 0) (in **d**). Analysis of the muscle-specific functional outcome measure in participants (MRC score), showing slightly better MRC scores in the VL muscles. p-values depict the results of a Fisher's exact test for normal MRC score (score = 5) versus reduced MRC score (score < 5). **f-g)** Analysis of molecular signature expression (DUX4 signature **(f)** and PAX7 score **(g)**) in the individual muscle types, showing similar patterns between control and FSHD biopsies. p-values depict the results of a Student's t-test. **h-j)** Paired analysis of nine muscle biopsy pairs (from all nine FSHD participants that donated a biopsy from both the VL and TA muscle) for muscle pathology (histology sum score (**h**)), DUX4 signature expression (**i**) and PAX7 scores (**j**). p-values depict the results of a paired Student's t-test. p-values: ns = not significant, * = p < 0.05, ** = p < 0.01, *** = p < 0.001, **** = p < 0.0001. All data plots are generated in R (v4.0.3, www.R-project.org) using the package *ggplot2* (v3.3.3). Figure and panel layout was further adapted in Adobe illustrator CC 2018 (www.adobe.com).

Supplementary Figure S6

Facioscapulohumeral dystrophy transcriptome signatures correlate with different stages of disease and are marked by different MRI biomarkers

Van den Heuvel, A & Lassche, S et al

submission ID: 631a14da-1e22-4289-88d4-f5e7e5973f60

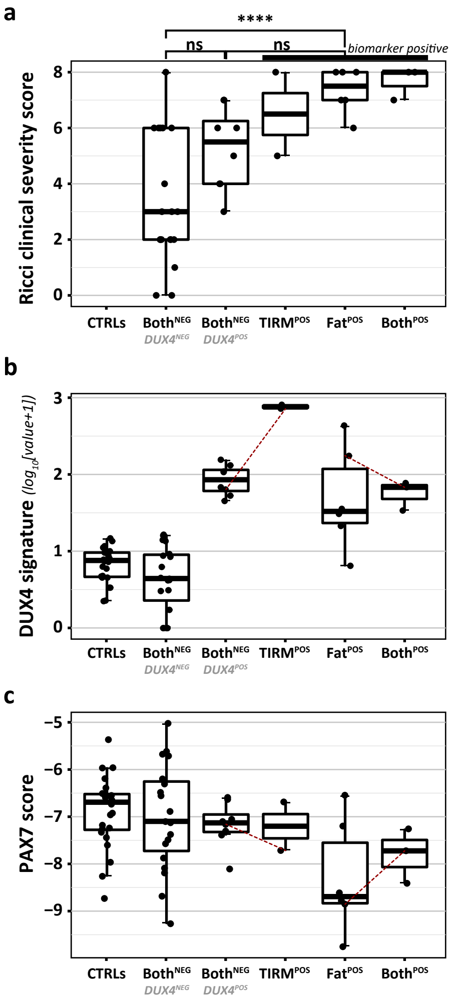

Supplementary Figure S6. CSS and signature expression in all individual subgroups.

a) Ricci clinical severity scores for all included participants, separated based on imaging biomarker signal and DUX4 signature expression in the imaging biomarker-negative subgroup. **b)** DUX4 signature expression in all subgroups as in **a**. **c)** PAX7 score in all subgroups as in **a**. The red dashed lines connect the paired muscle biopsies taken from the same muscle. All data plots are generated in R (v4.0.3, www.R-project.org) using the package *ggplot2* (v3.3.3). Figure and panel layout was further adapted in Adobe illustrator CC 2018 (www.adobe.com).

Supplementary Figure S7

Facioscapulohumeral dystrophy transcriptome signatures correlate with different stages of disease and are marked by different MRI biomarkers

Van den Heuvel, A & Lassche, S et al

submission ID: 631a14da-1e22-4289-88d4-f5e7e5973f60

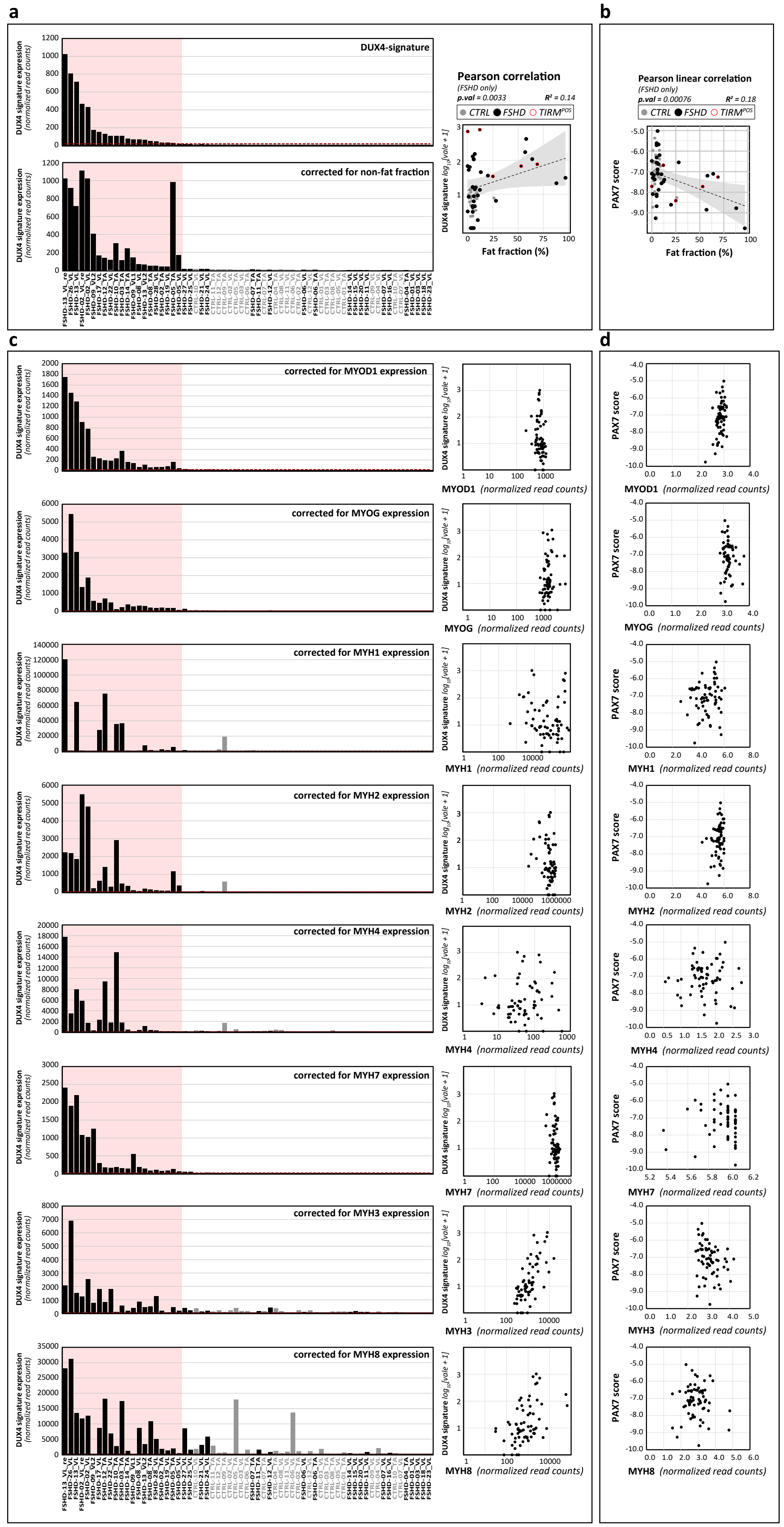

Supplementary Figure S7. FSHD signature corrections for muscle cell content.

a) DUX4 signature corrections based on fat fraction in the muscle. Top barplot; uncorrected scores (see also Supplemental Fig. S2). Bottom barplot; corrected scores. Scatterplot (right); correlation between DUX4 signature scores and fat fraction. **b)** Correlation between PAX7 scores and fat fraction. In both scatterplots in **a-b)** The linear regression line is based on the FSHD samples only. Grey shadings depict the 95%-confidence interval of the linear regession line. TIRM^POS biopsies are highlighted with a red outline for reference. **c)** DUX4 signature score corrections based on myogenic marker expression. Barplots; corrected scores. Scatterplots; correlation between DUX4 signature scores and myogenic marker gene expression levels. Biopsies originally classified DUX4^POS are highlighted with red shadings in the barplots. For best comparison with the DUX4 signature score, myogenic marker gene expression was first sequence depth-normalized following the median of ratios method implemented in DESeq2 R Package (v1.24.0) **d)** Correlation between PAX7 scores and myogenic marker gene expression levels. For best comparison with the PAX7 score, myogenic marker gene expression was first log-transformed (log_{10}[value+1]) and quantile-normalized. All plots are generated in Microsoft Excel, except for the scatterplots in a-b, which are generated in R (v4.0.3, www.R-project.org) using the packages *ggplot2* (v3.3.3) and *ggpubr* (v0.4.0). Figure and panel layout was further adapted in Adobe Illustrator CC 2018 (www.adobe.com).

Supplementary Figure S8

Facioscapulohumeral dystrophy transcriptome signatures correlate with different stages of disease and are marked by different MRI biomarkers

Van den Heuvel, A & Lassche, S et al

submission ID: 631a14da-1e22-4289-88d4-f5e7e5973f60

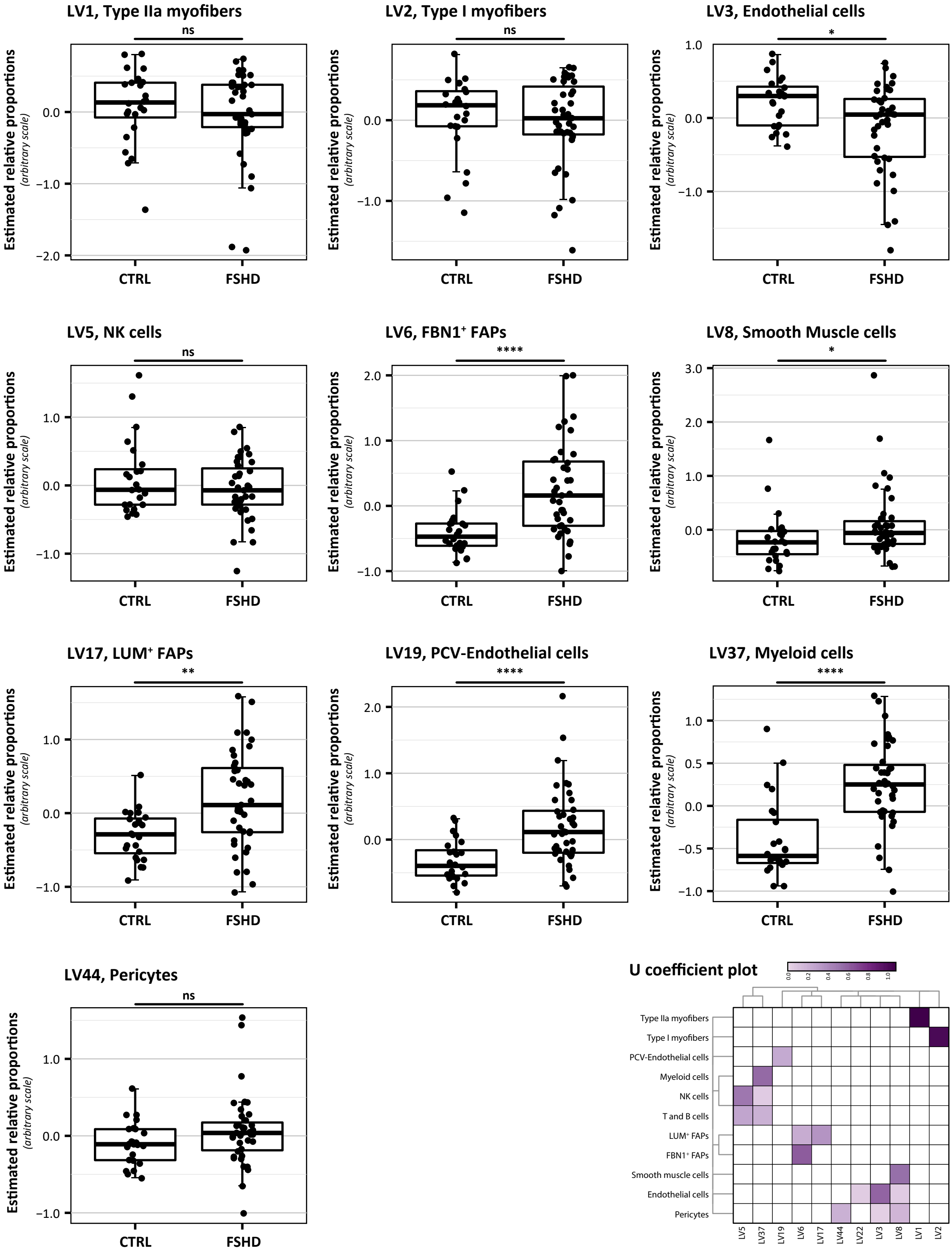

Supplementary Figure S8. Estimated relative contributions of all identified muscle and non-muscle cell types in FSHD versus control muscle biopsies.
Results for all cell types previously identified by Rubenstein AB et al. (Rubenstein AB et al. Sci Rep 10, 229 (2020)) in healthy human muscle biopsies, for which a high-confidence representative latent vector could be identified in our PLIER analysis (see Methods for details on PLIER analysis). The U coefficient plot for the LV-cell type signature relation is plotted at the bottom-right of this figure. This excluded *satellite cells* as no high-confidence representative LV for this cell type could be detected in our data. In addition, the *B and T cells* signature overlapped with *LV5, NK cells* and *LV37, Myeloid cells*, suggesting that distributions of these cell types cannot be discriminated. Finally, *LV22, Endothelial cells* was excluded from the analysis due to its overlap with other cells types and its low U coefficient as compared to *LV3, Endothelial cells*. LV: latent vector best representing the respective cell type signature noted behind the LV number. NK cells: Natural killer cells. FBN1⁺ FAPs: Fibrilin-1 positive fibro-adipogenic progenitors. LUM⁺ FAPs: lumican-positive fibro-adipogenic progenitors. PCV-Endothelial cells: post-capillary venules endothelial cells. p-values depict the result of Mann-Whitney U test. p-values: ns = not significant, * = $p < 0.05$, ** = $p < 0.01$, *** = $p < 0.001$, **** = $p < 0.0001$. All data plots are generated in R (v4.0.3, www.R-project.org) using the package *ggplot2* (v3.3.3). The U coefficient plot is generated in R using the package *PLIER* (v0.99.0). Figure and panel layout was further adapted in Adobe illustrator CC 2018 (www.adobe.com).

Supplementary Figure S9

Facioscapulohumeral dystrophy transcriptome signatures correlate with different stages of disease and are marked by different MRI biomarkers

Van den Heuvel, A & Lassche, S et al

submission ID: 631a14da-1e22-4289-88d4-f5e7e5973f60

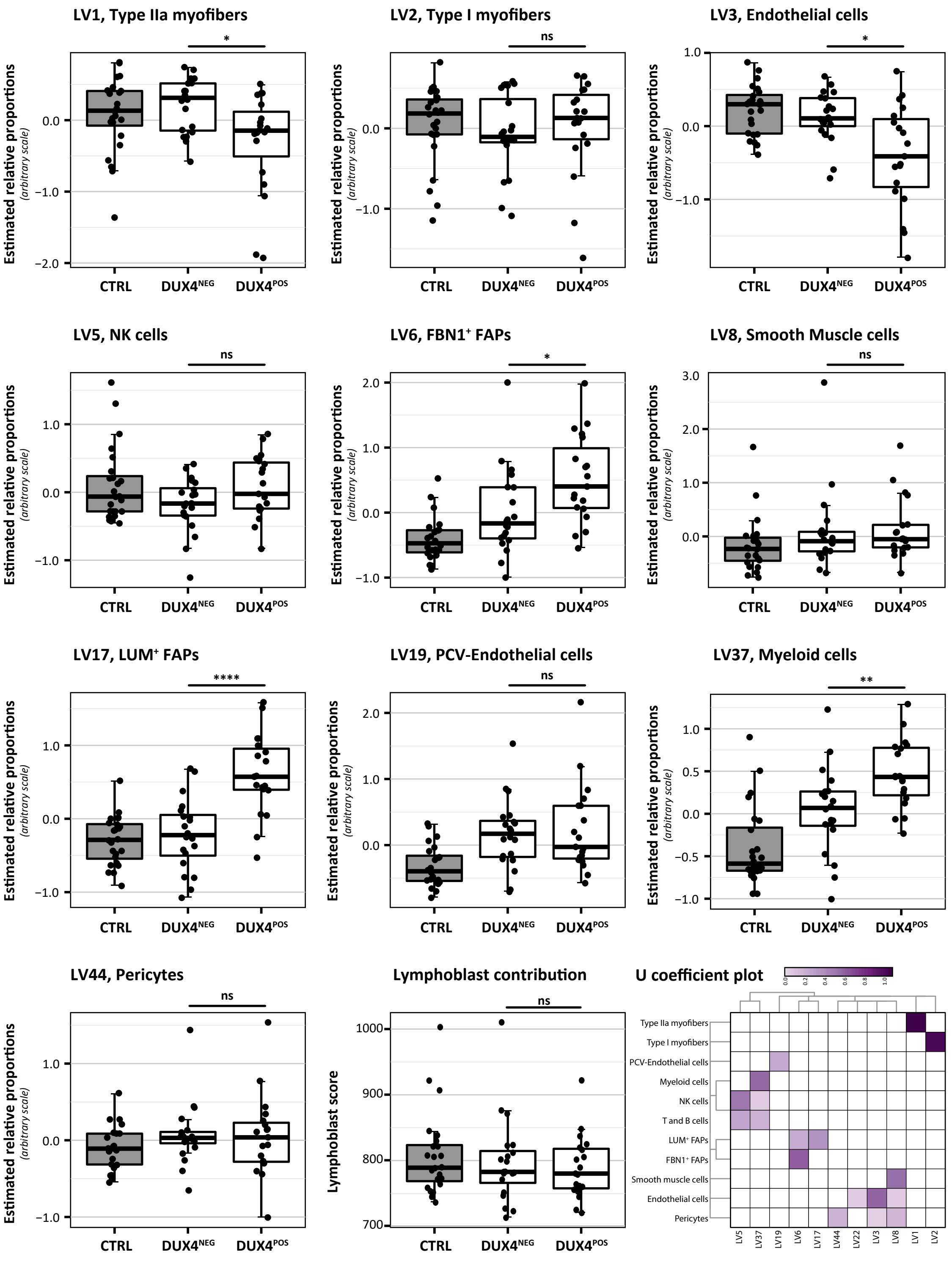

Supplementary Figure S9. Estimated relative contributions for all identified muscle and non-muscle cell types in DUX4^{POS} versus DUX4^{NEG} FSHD muscle biopsies. Results of our PLIER analysis as in Supplementary Fig. S8, but now for DUX4^{POS} versus DUX4^{NEG} FSHD muscle biopsies. The same U coefficient plot for the LV-cell type signature relation as in Supplementary Fig. S8 is plotted at the bottom-right of this figure for reference. The additional analysis for the contribution of lymphoblasts in DUX4 signature expression is added in the bottom middle panel. LV: latent vector best representing the respective cell type signature noted behind the LV number. NK cells: Natural killer cells. FBN1^{+} FAPs: Fibrilin-1 positive fibro-adipogenic progenitors. LUM^{+} FAPs: lumican-positive fibro-adipogenic progenitors. PCV-Endothelial cells: post-capillary venules endothelial cells. p-values depict the results of Mann-Whitney U tests. p-values: ns = not significant, * = $p < 0.05$, ** = $p < 0.01$, *** = $p < 0.001$, **** = $p < 0.0001$. All data plots are generated in R (v4.0.3, www.R-project.org) using the package *ggplot2* (v3.3.3). The U coefficient plot is generated in R using the package *PLIER* (v0.99.0). Figure and panel layout was further adapted in Adobe illustrator CC 2018 (www.adobe.com).

Supplementary Figure S10

Facioscapulohumeral dystrophy transcriptome signatures correlate with different stages of disease and are marked by different MRI biomarkers

Van den Heuvel, A & Lassche, S et al

submission ID: 631a14da-1e22-4289-88d4-f5e7e5973f60

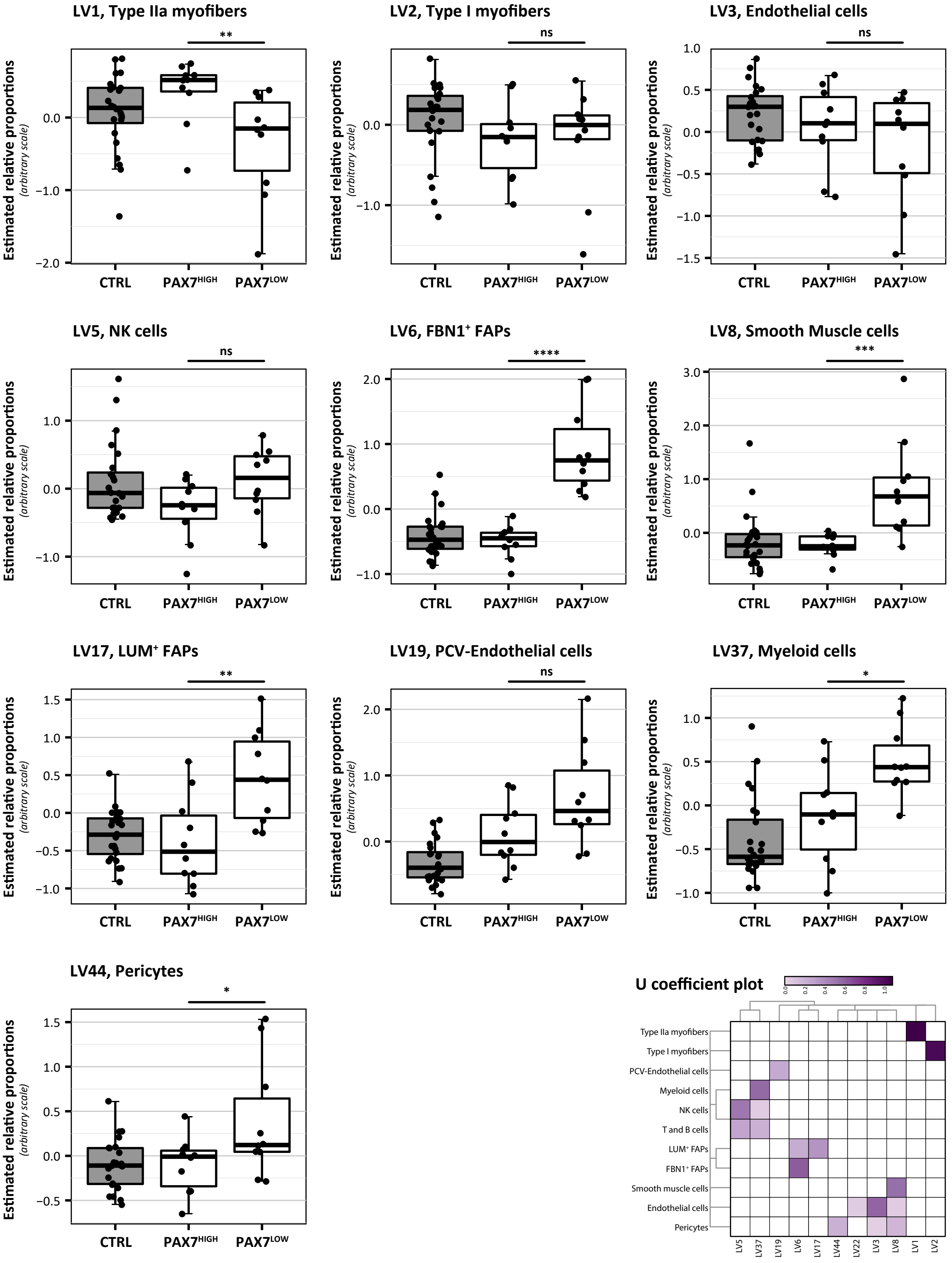

Supplementary Figure S10. Estimated relative contributions of all identified muscle and non-muscle cell types in $PAX7^{LOW}$ versus $PAX7^{HIGH}$ FSHD muscle biopsies. Results of our PLIER analysis as in Supplementary Fig. S8, but now for $PAX7^{LOW}$ versus $PAX7^{HIGH}$ FSHD muscle biopsies. The same U coefficient plot for the LV-cell type signature relation as in Supplementary Fig. S8 is plotted at the bottom-right of this figure for reference. LV: latent vector best representing the respective cell type signature noted behind the LV number. NK cells: Natural killer cells. $FBN1^+$ FAPs: Fibrilin-1 positive fibro-adipogenic progenitors. LUM^+ FAPs: lumican-positive fibro-adipogenic progenitors. PCV-Endothelial cells: post-capillary venules endothelial cells. p-values depict the results of Mann-Whitney U tests. p-values: ns = not significant, * = $p < 0.05$, ** = $p < 0.01$, *** = $p < 0.001$, **** = $p < 0.0001$. All data plots are generated in R (v4.0.3, www.R-project.org) using the package *ggplot2* (v3.3.3). The U coefficient plot is generated in R using the package *PLIER* (v0.99.0). Figure and panel layout was further adapted in Adobe illustrator CC 2018 (www.adobe.com).

Supplementary Figure S11

Facioscapulohumeral dystrophy transcriptome signatures correlate with different stages of disease and are marked by different MRI biomarkers

Van den Heuvel, A & Lassche, S et al

submission ID: 631a14da-1e22-4289-88d4-f5e7e5973f60

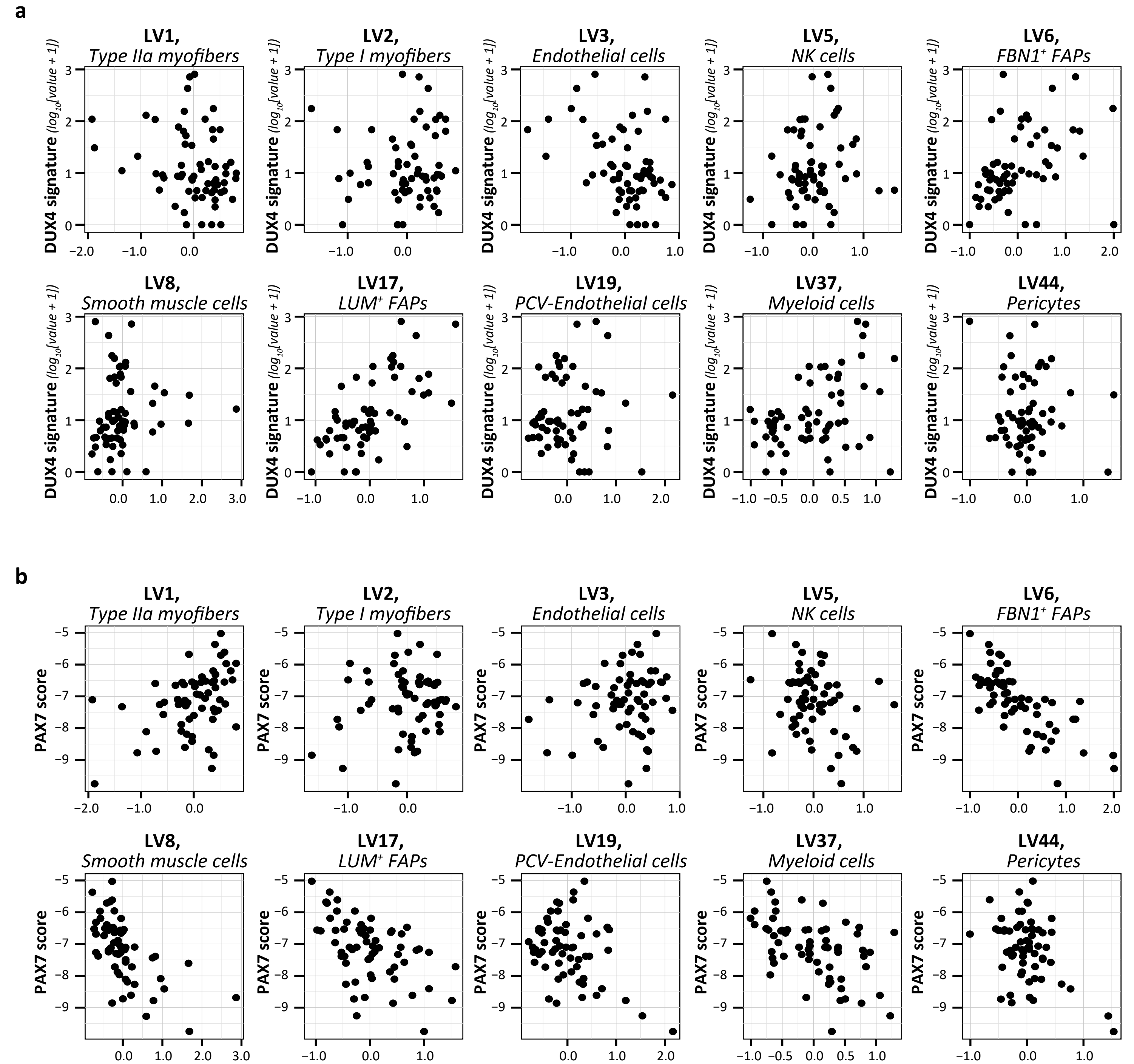

Supplementary Figure S11. Correlations between the estimated relative contributions of all identified muscle and non-muscle cell types and both FSHD expression signatures.

a) Results of our PLIER analysis as in Supplementary Fig. S8, but now showing the quantitative correlations with DUX4 signature expression levels. **b)** Results of our PLIER analysis as in Supplementary Fig. S8, but now showing the quantitative correlations with PAX7 scores. LV: latent vector best representing the respective cell type signature noted behind the LV number. NK cells: Natural killer cells. FBN1^{+} FAPs: Fibrilin-1 positive fibro-adipogenic progenitors. LUM^{+} FAPs: lumican-positive fibro-adipogenic progenitors. PCV-Endothelial cells: post-capillary venules endothelial cells. All plots are generated in Microsoft Excel and are based on the estimated cell type proportions calculated in R using the package *PLIER* (v0.99.0). Figure and panel layout was further adapted in Adobe illustrator CC 2018 (www.adobe.com).
